# Dachaihu decoction alleviates septic liver injury by modulating the intestinal barrier dysfunction and suppressing the NF-κB/NLRP3/Caspase-1 signaling pathway

**DOI:** 10.1186/s13020-026-01420-1

**Published:** 2026-06-16

**Authors:** Zhen Yang, Xingyu Kao, Lin Zhang, Na Huang, Yi Wang, Jingli Chen, Mingfeng He, Qi Tang, Zhangrong Liang

**Affiliations:** 1 The Eighth Clinical Medical College of Guangzhou University of Chinese Medicine, Foshan, 52800 China; 2 Foshan Hospital of Traditional Chinese Medicine, Foshan, 52800 China; 3 Department of Cardiovascular, Integrated Hospital of Traditional Chinese Medicine, Southern Medical University, Guangzhou, 510315 China

**Keywords:** Dachaihu decoction, Septic liver injury, Gut-liver axis, Gut microbiota, Inflammation

## Abstract

**Background:**

Intestinal barrier dysfunction is a key driver of septic liver injury (SLI). Dachaihu decoction (DCHD), a classic traditional Chinese medicine formula recorded in *the Treatise on Cold Damage*, is widely used to treat gastrointestinal and hepatic inflammatory conditions. The primary objective of our research was to elucidate the protective effects of DCHD against SLI and the underlying molecular mechanisms.

**Methods:**

In a murine model of sepsis induced by cecal ligation and puncture (CLP), we evaluated the therapeutic effects of DCHD on SLI by assessing serum liver enzymes, histopathology, oxidative stress, hepatocyte apoptosis, and inflammatory cytokines. Intestinal barrier integrity was examined via transmission electron microscopy, serum biomarkers (D-lactate, DAO, LPS), and tight junction proteins (ZO-1, Occludin, E-cadherin). Gut microbiota composition was analyzed using 16S rRNA sequencing. Chemical profiling of DCHD was performed via UPLC-Q-TOF–MS. Integrated network pharmacology, bioinformatics, and transcriptomic analyses identified the NF-κB/NLRP3/Caspase-1 axis as a potential mechanism, which was validated in vivo and in LPS-stimulated immortalized mouse Kupffer cells (ImKCs). Functional involvement of TLR4 and NLRP3 was further confirmed by genetic silencing of TLR4 with siRNA and pharmacological inhibition using TAK-242 (TLR4 inhibitor) and MCC950 (NLRP3 inhibitor).

**Results:**

DCHD treatment attenuated liver injury in CLP-induced septic mice, as evidenced by improved liver function, attenuated histopathology, reduced oxidative stress, suppressed inflammation, and decreased hepatocyte apoptosis. These hepatoprotective effects were associated with reduced intestinal permeability and enhanced barrier integrity, alongside gut microbiota remodeling characterized by enrichment of beneficial bacteria and reduced abundance of gram-negative genera (e.g., *Klebsiella*, *Enterobacter*, *Proteus*), leading to decreased LPS production and translocation to the liver. Integrated network pharmacology and transcriptomics revealed the NF-κB/NLRP3/Caspase-1 axis as a central mechanism, with DCHD downregulating p-p65, p-IκBα, NLRP3, ASC, and Cleaved Caspase-1 in vivo and in LPS-stimulated ImKCs. Functional validation using TLR4 siRNA and the inhibitors TAK-242 and MCC950 confirmed that DCHD might attenuate liver inflammatory injury primarily through the NF-κB/NLRP3/Caspase-1 signaling pathway.

**Conclusion:**

DCHD may alleviate SLI by enhancing the intestinal barrier, potentially reducing the translocation of gut-derived LPS to the liver, and subsequently inhibiting the NF-κB/NLRP3/Caspase-1 axis, highlighting its considerable translational potential for SLI therapy.

**Graphical abstract:**

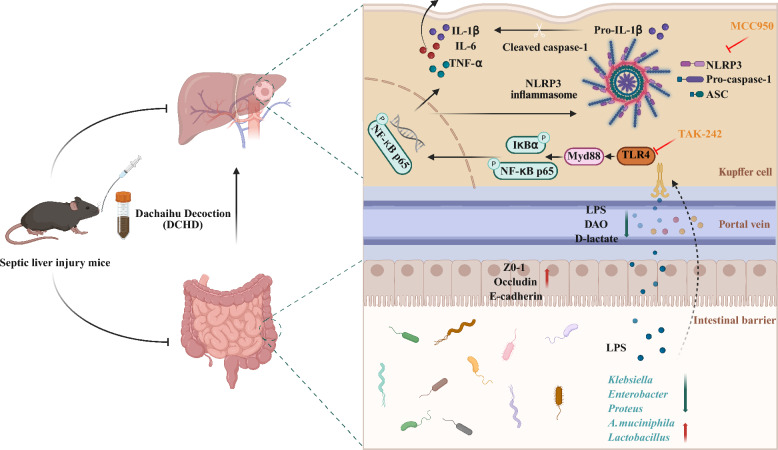

**Supplementary Information:**

The online version contains supplementary material available at 10.1186/s13020-026-01420-1.

## Introduction

Sepsis is a life-threatening syndrome triggered by a dysregulated host response to infection that can lead to multiple organ dysfunction syndrome (MODS). It is a prevalent high-risk complication among critically ill patients, such as those with burns, trauma, or post-operative conditions [1]. Sepsis is characterized by rapid progression and high mortality rate. Globally, an estimated 48.9 million people develop sepsis annually, resulting in 11.0 million deaths. This accounts for 19.7% of all global deaths, presenting a formidable challenge to health systems worldwide [2]. The liver serves as a critical effector in maintaining host homeostasis and orchestrating defense mechanisms, constituting a vital barrier against microbial invasion, and plays an indispensable role in preserving internal environmental stability; however, it is highly susceptible to infection or drug-induced injury [3]. Among sepsis-associated organ injuries, liver is one of the most vulnerable targets, with liver injury manifesting in approximately 34.7% of septic patients [4]. Septic liver injury (SLI) typically develops from active hepatocyte dysfunction to liver injury, followed by persistent inflammation, perfusion insufficiency, and finally, liver failure [5]. Of particular concern, the mortality rate of septic patients with liver dysfunction exceeds that of sepsis-associated lung injury by 54%, which markedly impairing clinical outcomes [6]. The precise pathogenesis of SLI remains unclear and effective therapeutic interventions are lacking. Current understanding implicates contributing mechanisms, such as cytokine storms, direct endotoxin toxicity, hepatic microcirculatory dysfunction, and bilirubin-bile acid dysregulation [7]. Consequently, delving deeper into the pathophysiological mechanisms underlying SLI and exploring novel diagnostic and therapeutic strategies represents an urgent scientific imperative and major research focus.

The intestinal barrier constitutes a multifaceted defense system against pathogen translocation, and is primarily composed of microbial, chemical, mechanical, and immune barriers [8]. Its integrity serves as the core protective mechanism preventing the mass translocation of gut-derived toxins into portal circulation during sepsis, thereby averting excessive immuno-inflammatory assault in the liver [9]. Notably, intestinal flora dysbiosis is a pivotal factor in precipitating intestinal barrier compromise (“leaky gut”), thereby serving as an initiating or exacerbating factor in the pathogenesis of diverse hepatobiliary pathologies [10]. Moreover, adjacent epithelial cells establish robust intercellular adhesion via tight junction complexes, which are indispensable for preserving gut barrier integrity [11, 12]. During sepsis, the disruption of the intestinal barrier by inflammatory triggers initiates a cascade of increased permeability and ecological imbalances, predisposing the host to gut dysbiosis. Consequently, gut-derived flora and their metabolites can gain access to the liver via the portal or biliary system, triggering an inappropriate immune response or severe hepatic inflammation [13]. The release of intestinal lipopolysaccharide (LPS) into the bloodstream can induce hepatic inflammation through the activation of the toll-like receptor-4 (TLR4)-mediated nuclear factor-κB (NF-κB) signaling pathway and NOD-like receptor protein 3 (NLRP3) inflammasome [14].

Traditional Chinese Medicine (TCM) has shown unique therapeutic benefits in addressing sepsis and related MODS, particularly for liver injury [15, 16]. Dachaihu decoction (DCHD) is originally records in *the Treatise on Cold Damage*, written by Zhang Zhongjing in the Eastern Han Dynasty, exerts effects of “reconciling Shaoyang and clearing Yang Ming evil heat” based on the TCM theory, and has been chosen to treat various liver disorders in clinical practice for a long time [17, 18]. DCHD is composed of eight herbs, namely, *Bupleurum chinense* DC. (Chaihu), *Scutellaria baicalensis* Georgi (Huangqin), *Pinellia ternata* Breit. (Banxia), *Citrus aurantium* L. (Zhishi), *Rheum officinale* Baill*.* (Dahuang), *Zingiber officinale* Roscoe. (Shengjiang), *Paeonia lactiflora* Pall. (Shaoyao), *Ziziphus jujuba* Mill. (Dazao), which work together to exert the effects of dredging the fu-organs and draining heat (*tong fu xie re*), as well as soothing the liver and promoting bile flow (*shu gan li dan*). DCHD has been shown to have various pharmacological effects, including regulation of intestinal flora [17], anti-inflammatory [18, 19], inhibition of macrophage infiltration [19], prevention of pancreatic fibrosis [20].

Our previous clinical study demonstrated that DCHD mitigated systemic inflammation and improved liver and gastrointestinal functions in patients with SLI, thereby effectively mitigating sepsis-induced MODS [21]. However, the mechanism by which DCHD ameliorates SLI remains unclear. We hypothesized that DCHD could alleviate SLI by repairing intestinal barrier dysfunction, reducing the translocation of gut-derived LPS into the liver, and subsequently inhibiting the activation of NF-κB/NLRP3/Caspase-1 signaling pathway. First, a murine model of sepsis was established using cecal ligation and puncture (CLP) method. Furthermore, we employed an integrated strategy combining network pharmacology, bioinformatics, 16S rRNA sequencing, hepatic transcriptomics, and experimental validation to investigate the protective role of DCHD against SLI and elucidate its underlying mechanisms in vivo and in vitro. Collectively, our research not only exemplifies the holistic concept of TCM but also lays a foundation for innovative therapeutic paradigms against SLI.

## Materials and methods

### Preparation of DCHD and DCHD drug containing serum (DCHD-DS)

The ratio and decoction method for preparing the DCHD were based on our previous study [22]. The crude extract was standardized to a concentration of 1 g/mL and maintained at 4 °C for future applications. The details of the ingredients of DCHD are presented in Table S1.

Drug-containing serum can simulate the in vivo environment, reflect the true efficacy of drugs after biotransformation, and is a more scientific research method that has been widely used in in vitro studies of traditional Chinese medicine compounds [23]. The DCHD-DS was prepared according to a previously established protocol [22]. Twenty male Sprague–Dawley (SD) rats (weighing 200 ± 20 g) were purchased from Guangzhou Regal Biotechnology Co., Ltd. (SCXK (Yue) 2021-0059, Guangzhou, China), After 3 days of acclimatization in a standard specific pathogen-free (SPF) laboratory environment, and randomly assigned to two groups: the control group (*n* = 8) and the DCHD-DS group (*n* = 12). Rats in the DCHD-DS group received twice-daily oral gavage of DCHD (7.56 g/kg/day, 1 ml/100 g) at 12-h intervals, while those in the control group were administered an equivalent volume of saline. The rats were anesthetized by intraperitoneal injection of 3% pentobarbital sodium (45 mg/kg) 1 h after the last gavage on the 3rd day, and blood was collected through the abdominal aorta and centrifuged after 2 h of clotting to obtain serum, which was aliquoted in a sterile environment. After inactivation (56 °C water bath, 30 min) and bacterial removal via 0.22 µm filtration, the serum samples were stored at − 80 °C until needed for in vitro studies [24].

### Ultra-high performance liquid chromatography with quadrupole time-of-flight mass spectrometry (UPLC-Q-TOF–MS) analysis

UPLC-Q-TOF–MS was employed to acquire mass spectrometric data from the DCHD samples, followed by comprehensive chemical profiling using a high-resolution mass spectrometry database dedicated to natural products. Separation was performed on a Waters CORTECS® UPLC® T3 column (2.1 × 100 mm, 1.6 µm, Waters Corporation, Milford, MA, USA). The separation was performed using a gradient elution program with acetonitrile (mobile phase A) and 0.1% aqueous formic acid (mobile phase B) at a flow rate of 0.3 mL/min. The column temperature was maintained at 30 °C and 2 µL of sample was injected. Detection was performed over the wavelength range of 190–400 nm. The gradient elution program was employed as follows: 0–2 min, 2% B; 2–20 min, 2% → 8% B; 20–30 min, 8% → 18% B; 30–35 min, 18% → 23% B; 35–40 min, 23% → 30% B; 40–50 min, 30% → 50% B; 50–55 min, 50% B; 55–55.5 min, 50% → 2% B; 55.5–62 min, 2% B. Detection was achieved using an AB Sciex Triple TOF® 4600 (AB Sciex, USA) in both positive and negative ionization modes. Key source parameters included: drying gas (N₂) temperature, 350 °C; nebulizer gas (N₂) pressure, 2.4 × 10^5^ Pa; drying gas (N₂) flow rate, 8 L/min; sheath gas temperature, 350 °C; sheath gas flow, 11 L/min; electrospray voltage, ± 3,500 V; capillary exit voltage, 150 V; nozzle voltage, 65 V; octopole RF voltage, 750 V. Full scan spectra were acquired over the *m/z* range 100–1000. Collision-induced dissociation (CID) experiments utilized stepped collision energies of 10 eV, 20 eV, and 40 eV. Data analysis and compound annotation were conducted using Thermo Xcalibur™ and Compound Discoverer software platforms, respectively.

### Network pharmacology and bioinformatics analysis

The core compounds of DCHD identified via UPLC-Q-TOF–MS analysis were compared with those in the TCMSP database (https://tcmsp-e.com/tcmsp.php) [25]. SwissTargetPrediction [26] (http://www.swisstargetprediction.ch/) was used to predict potential targets of the compounds. The “DCHD-active compounds-target” network was constructed by Cytoscape3.7.0. Subsequently, sepsis-related sequencing data for GSE54514, GSE57065, and GSE95233 were downloaded from the NCBI Gene Expression Omnibus [27] (GEO; https://www.ncbi.nlm.nih.gov/geo/). Differentially expressed genes (DEGs) were identified using the screening criteria of |log2 fold-change (FC)| ≥ 0.5 and a p-value < 0.05. The search terms “sepsis” and “liver injury” were used to search for disease targets related to SLI in GeneCards database (https://www.genecards.org/). Finally, we obtained the intersection of DCHD-related, sepsis-related, and liver injury-related targets, which were defined as common targets of DCHD against SLI.

Common targets were imported into the STRING database (version 11.5, https://string-db.org/) to construct a protein–protein interaction (PPI) network. The core targets were identified by intersecting the top 15 targets derived from the Maximal Clique Centrality (MCC), Degree, Edge Percolated Component (EPC), Maximum Neighborhood Component (MNC), and betweenness centrality algorithms using the CytoHubba plugin in Cytoscape 3.7.0.

Functional enrichment analysis was conducted using the DAVID database (https://david.abcc.ncifcrf.gov/), including Gene Ontology (GO) annotations and Kyoto Encyclopedia of Genes and Genomes (KEGG) pathways. Biological processes (BP), cellular components (CC), and molecular functions (MF) were the three main categories of GO analysis. Concurrently, KEGG pathway analysis revealed the critical signaling pathways of DCHD in SLI. Analytical processing and visualization were performed using a bioinformatics platform (https://www.bioinformatics.com.cn/).

### Molecular docking verification

The 3D molecular structures were procured from PubChem (https://pubchem.ncbi.nlm.nih.gov) by querying canonical compound names and assigning CAS registry numbers. These initial structures were subjected to energy minimization and optimization using Chem3D software. Molecular structures were subsequently constructed using ChemDraw v11.0, and exported in MOL2 format. Conversion to the PDBQT format was performed using MGLTools 1.5.6. The initial coordinates for the proteins of interest were obtained from the RCSB Protein Data Bank (PDB) database (http://www.rcsb.org/structure/) to serve as the structural basis for subsequent analysis. The protein structures were prepared in MGLTools 1.5.6, to add polar hydrogens, assign Gasteiger charges, and merge nonpolar hydrogens prior to export in the PDBQT format. Molecular docking simulations and visualization were performed using AutoDock 4.2 and BIOVIA Discovery Studio 2019. Potent receptor-ligand interactions were defined by binding energy thresholds ≤ − 4.0 kcal/mol, with a lower binding energy indicating greater complex stability [28]. To further investigate protein flexibility, normal mode analysis was performed using the iMODS platform (https://imods.iqf.csic.es/), which enables predictive modeling of conformational changes and dynamic properties. Subsequently, the directional motions of individual residues and collective dynamics of the protein are graphically represented. Deformability quantifies the magnitude of permissible elastic distortion for each amino acid residue, where higher peak values correspond to an enhanced propensity for local structural deformation. The B-factor is a measure of the thermal motion of the individual atoms in a protein. It quantifies the amplitude of the atomic positional fluctuations and serves as an indicator of positional uncertainty and flexibility.

### Animal model and treatment

6–8 weeks old C57BL/6 mice (male, weighing 20–22 g) were individually housed in a SPF level laboratory animal feeding room with a standard 12/12 h light/dark schedule (22–24 °C with 50–60% relative humidity) with lights on (150 lx intensity) at 06:00 a.m. Following a 3-day acclimatization period, all interventions and model establishment procedures were commenced.

In accordance with CLP-induced sepsis model protocol [29], the mice were anesthetized via an intraperitoneal injection of 1% pentobarbital sodium (0.08 mL/10 g) following a 12-h fast. A laparotomy was performed to expose the cecum. The distal portion of the cecum below the ileocecal valve was ligated using a 4–0 silk suture and subsequently punctured twice using a 21-gauge needle. Subsequently, a moderate amount of fecal content was gently squeezed from the punctured cecum and repositioned in the abdominal cavity for surgical closure. In the sham group, the cecum was exposed via laparotomy but neither ligated nor punctured before closure. For post-operative support, all mice were subcutaneously administered warm saline (0.5 mL/10 g) and placed on a heating pad until complete recovery. All the surgical procedures were performed by a single surgeon to ensure consistency. After 12 the operation, the mice were scored by two investigators. The sepsis score consists of seven components: appearance, activity, response to stimuli, level of consciousness, respiratory rate, eyes, and respiratory quality, with scores ranging from 0 to 4 [30]. Importantly, liver function and histopathological damage are critical indicators of the successful establishment of an SLI mouse model [31].

Prior to modeling, sixty mice were randomly divided into five groups: sham (*n* = 10), CLP (*n* = 14), and low-, medium-, and high-dose DCHD (*n* = 12). The dosage for oral gavage and the method of euthanasia in mice were performed as previously described in our study [22]. Following surgery until recovery from anesthesia (approximately 2 h), mice in the DCHD-L, DCHD-M, and DCHD-H groups were administered DCHD via oral gavage at doses of 5.46, 10.92, and 21.84 g/kg/d, respectively, twice daily at 12-h intervals. The dosage was computed by converting the human equivalent dose (84 g crude herbs/70 kg/day) via body surface area conversion (0.0026) [32]. Mice in the CLP and sham groups were intragastrically administered an equivalent volume of saline solution. Finally, all the mice were sacrificed 24 h post-surgery, with no animals excluded during this period [33]. Blood and colonic, fecal, and liver tissue samples were systematically collected, and mice that died before the 24-h endpoint were not included in the biochemical analyses. The 24 h survival rate and murine sepsis score (MSS) were concurrently documented.

### Biochemical index and cytokine measurement

Serum alanine aminotransferase (ALT) and aspartate aminotransferase (AST) levels were quantified using commercial assay kits (Changchun Huili Biotechnology Co., Changchun, China) following the manufacturer’s protocol. The activities of superoxide dismutase (SOD), malondialdehyde (MDA), catalase (CAT), and glutathione peroxidase (GSH-Px) were determined using commercial assay kits according to the manufacturer’s protocols (A001-3-2, A003-1-2, A007-1-1, and A005-1-2; Nanjing Jiancheng Bioengineering Institute, Nanjing, China). The serum concentrations of interleukin-6 (IL-6) (RK00008; ABclonal, Wuhan, China), tumor necrosis factor-α (TNF-α) (RK00027; ABclonal), IL-1β (RK00006; ABclonal), D-lactate (CB12314-Mu; Mlbio, Shanghai, China), and diamine oxidase (DAO) (MM-0228M1, Mlbio) in mice were quantified using commercial ELISA kits. An LPS ELISA kit (CSB-E13066m; Cusabio, Wuhan, China) was used to detect the concentration of LPS in serum and liver tissues, and the absorbance was measured using a spectrophotometer (BioTek, USA).

### Histopathologic analysis and immunohistochemistry (IHC) analysis

Following fixation in an adequate volume of 4% paraformaldehyde (PFA) for over 48 h, liver tissues were dehydrated using a graded ethanol series and subsequently embedded in paraffin. Following preparation of 4 μm-thick sections, hematoxylin and eosin (H&E) staining was performed to facilitate histopathological examination under a light microscope (Carl Zeiss, Germany). Histopathological scoring of the liver injury was performed according to previously described criteria. Hepatic damage was quantified on a summative scale that integrated three independent pathological parameters: inflammation, thrombus formation, and necrosis. Each parameter was scored on a scale of 0 to 4, with 0 indicating normal tissue and 4 indicating severe injury [34].

IHC analysis was performed to assess the expression of TLR4 (66,350—1-Ig; Proteintech, Wuhan, China), MyD88 (67969-1-Ig; Proteintech), NF-κB p65 (ab32536; Abcam, USA), F4/80 (ab300421; Abcam), and CD11b (ab133357; Abcam) in liver tissues. Formalin-fixed, paraffin-embedded liver sections were subjected to standard processing including deparaffinization and heat-induced antigen retrieval. Subsequently, non-specific binding sites were blocked to minimize background interference. Following primary antibody application at 4 °C overnight, the sections were subjected to a 50-min incubation with the corresponding secondary antibodies at 37 °C. Subsequent visualization was performed using diaminobenzidine (DAB) chromogen development and hematoxylin counterstaining. Tissue sections were finalized by dehydration, clearing, and cover-slipping. The acquired microscopic images were quantified using ImageJ software (National Institutes of Health, USA).

### Terminal deoxynucleotidyl transferase dUTP nick end labeling (TUNEL) staining

The TUNEL assay (E-CK-A320, Elabscience, China) was employed to determine hepatocyte apoptosis, following the supplier's instructions. Tissue sections were processed with Proteinase K (37 °C, 15 min), washed with PBS, and incubated with the TUNEL reagent in darkness. Nuclei were counterstained with 4',6-diamidino-2-phenylindole (DAPI), and the slides were preserved with an anti-fade mounting medium. Fluorescent signals were captured using a Zeiss Axio Scope A1 microscope (Carl Zeiss, Germany), with DAPI-stained nuclei appearing blue and TUNEL-positive apoptotic nuclei fluorescing green under appropriate excitation. The apoptosis rate (apoptotic cells/total cells) was calculated by image analysis using the ImageJ software.

### ROS assay

Cryosections (8–10 μm) of snap-frozen murine liver tissue embedded in OCT compound were prepared using a cryostat. Sections were incubated with 10 μM dihydroethidium (DHE, s0063, Beyotime, China) at 37 °C for 30 min in the dark. DHE is oxidized by superoxide to form red-fluorescent ethidium. After staining, the sections were washed with PBS and mounted with anti-fade medium. Imaging was performed using a confocal microscope with appropriate filters. The fluorescence intensity was quantified using ImageJ software.

### Transmission electron microscopy (TEM)

Freshly isolated colon tissues were promptly minced (1 mm^3^ fragments) and subjected to immersion fixation in 2.5% glutaraldehyde (BL911A; Biosharp, Hefei, China). primary fixation was performed at room temperature under light-protected conditions for 2 h, with subsequent storage at 4 °C. The samples were post-fixed in 1% osmium tetroxide, followed by routine dehydration using a graded ethanol and acetone series. Following dehydration, the samples were infiltrated and cast into blocks of polymerized epoxy resin. The resin blocks were polymerized by heating overnight. Ultrathin sections were generated using an ultramicrotome, subjected to double staining with lead citrate and uranyl acetate, and subsequently imaged using TEM (HT7800, Hitachi, Japan). Representative images were captured to assess the ultrastructural alterations in the colon. Based on these images, the colonic microstructural damage was quantified using an established scoring system [35].

### 16S rRNA sequencing

Total nucleic acids were extracted from murine fecal samples using a commercial kit (DP812; Tiangen Biotech, Beijing, China) as recommended by the manufacturer. Subsequent procedures, including sample processing, nucleic acid quantification, amplification, and SMRTbell library construction, followed established methodologies [36]. Briefly, the nucleic acid concentrations were quantified using a microplate reader (GeneCompang Limited, Synergy HTX). The integrity of the amplified PCR products was verified using 1.8% agarose gel electrophoresis. Full-length 16S rRNA genes were amplified using universal primers 27F_(16S-F) (5′-AGRGTTTGATYNTGGCTCAG-3′) and 1492R_(16S-R) (5′-TASGGHTACCTTGTTASGACTT-3′). The library was prepared and quality-controlled prior to sequencing on a PacBio Sequel II system (Pacific Biosciences, CA, USA) (Supplementary Material 1).

Raw sequences were processed in QIIME2 using the DADA2 pipeline for quality filtering, denoising, paired-end merging, and chimera removal to yield amplicon sequence variants (ASVs). Taxonomic profiles were generated by assigning ASVs to phylum, genus, and species levels. The differential abundance between the groups was assessed by analyzing the composition of the microbiomes (ANCOM). Biomarker taxa were identified via Linear Discriminant Analysis Effect Size (LEfSe), with a significance threshold of LDA score > 4. Venn diagrams were constructed to delineate unique and shared ASVs across the sample groups. α-Diversity indices were calculated using the QIIME2 diversity plugin, and intergroup differences were evaluated using the Wilcox Test. β-Diversity was analyzed by Partial Least Squares Discriminant Analysis (PLS-DA) using the mixOmics R package.

Finally, the association between specific intestinal bacterial genera and serum LPS levels was evaluated using the Spearman's correlation analysis.

### Hepatic transcriptomic analysis

Following extraction from mouse liver tissues using TRIzol (Thermo Fisher Scientific, USA), the integrity of the total RNA was assessed using an Agilent Bioanalyzer 2100 (Agilent Technologies, USA). Prior to sequencing, libraries were prepared using the NEBNext® Ultra™ RNA Library Prep Kit for Illumina® (NEB, USA) following the manufacturer's guidelines. Upon purification of the PCR products via the AMPure XP system and library qualification, sequencing was conducted on an Illumina NovaSeq platform, generating 150 bp paired-end reads. These clean reads were mapped to the reference genome using Hisat2 v2.0.5. The assembly of novel transcripts was accomplished using StringTie (v1.3.3b) and gene expression levels were quantified using FPKM. Differential expression analysis was performed using DESeq2, with significance defined as a *p*-value < 0.05 and a |log_2_ fold change|> 1. A PPI network was constructed based on the DEGs, followed by GO and KEGG enrichment analyses, to further investigate their functional implications.

### Cell culture and treatment

Immortalized Mouse Kupffer cells (ImKCs) (BNCC340733) were procured from the Cell Bank of Procell Life Science&Technology Co. (Wuhan, China) and used to establish a cell model of liver inflammatory damage. ImKCs were maintained in Dulbecco’s modified Eagle’s medium (DMEM; GIBCO BRL, Grand Island, NY, USA) supplemented with 10% fetal bovine serum (FBS; GIBCO, Grand Island, NY, USA) in a humidified 5% CO_2_ incubator (Thermo, USA) at 37 °C. ImKCs in good condition were treated with lipopolysaccharide LPS (L2880, 055:B5; Sigma, USA). ImKCs were seeded in 96-well plates at a density of 6 × 10^3^ cells/well for subsequent viability assessment using the CCK-8 assay (E-CK-A362; Elabscience, China). Upon reaching 60–70% confluence, the cells were exposed to increasing concentrations of LPS (0, 0.1, 1, 5, 10, and 100 µg/mL) or DCHD-DS (0, 5%, 10%, 15%, 20%, and 30%) for 24 h to evaluate cell viability.

To determine the contribution of the TLR4/NF-κB/NLRP3/Caspase-1 signaling pathway to DCHD-mediated anti-inflammatory effects in ImKCs, cells were incubated for 2 h with either the TLR4 inhibitor TAK-242 (resatorvid, 10 μM) (HY-11109; MedChemExpress, USA) [37, 38] or the NLRP3 inhibitor MCC950 (10 µM) (HY-12815; MedChemExpress, USA) [39] and then exposed to either LPS alone or in combination with 10% DCHD-DS. For the TAK-242 experiments, the five groups were: (1) control (CON), (2) LPS, (3) LPS + 10% DCHD-DS, (4) LPS + TAK-242; (5) LPS + TAK-242 + 10% DCHD-DS. For the MCC950 experiments, the groups were: (1) CON, (2) LPS, (3) LPS + 10% DCHD-DS, (4) LPS + MCC950, and (5) LPS + MCC950 + 10% DCHD-DS. To exclude the interference of the serum matrix, blank rat serum was used to adjust the volume to ensure that the final total serum concentration in all groups was consistent at 10%. After 24 h of incubation, the cellular samples were collected for subsequent analyses.

### RNA extraction and quantitative real-time PCR (qRT-PCR)

Total RNA was extracted from the liver tissues and ImKCs using TRIzol reagent (15,596,026; Invitrogen, USA). RNA purity and concentration were determined using a microplate reader (BioTek Epoch 2; Winooski, VT, USA). Complementary DNA was synthesized from mRNA following the manufacturer’s protocol for the reverse transcription kit (RR037A;, Takara, Japan) and subsequently stored at -80 °C. mRNA expression levels were measured using SYBR™ Select Master Mix (4,472,908; Thermo Fisher Scientific, USA) on an Applied Biosystems 7500 Fast Real-Time PCR System. The thermal cycling protocol was as follows: initial denaturation at 95 °C for 30 s, followed by 40 cycles of denaturation at 95 °C for 5 s and annealing/extension at 60 °C for 34 s. The relative mRNA expression levels were quantified using the 2^−ΔΔ^CT method. All primer sequences used in this study were synthesized by Shanghai Shenggong Biological Engineering Technology Co. Ltd. (Table 1).

**Table 1:** Primer sequences for qRT-PCR

Genes	Forward 5′–3′	Reverse 5′–3′
*TNF-α (Mouse)*	CCTGTAGCCCACGTCGTAG	GGGAGTAGACAAGGTACAACCC
*IL-1β (Mouse)*	GAAATGCCACCTTTTGACAGTG	TGGATGCTCTCATCAGGACAG
*IL-6 (Mouse)*	TAGTCCTTCCTACCCCAATTTCC	TTGGTCCTTAGCCACTCCTTC
*CXCL2 (Mouse)*	GGAAGCCTGGATCGTACCTG	TGAAAGCCATCCGACTGCAT
*MCP-1 (Mouse)*	CAGGTCCCTGTCATGCTTCT	GTGGGGCGTTAACTGCATCT
*CCL3 (Mouse)*	CCATATGGAGCTGACACCCC	GAGCAAAGGCTGCTGGTTTC
*IκBα (Mouse)*	CGAGACTTTCGAGGAAATACCC	GTCTGCGTCAAGACTGCTACA
*NF-κB (Mouse)*	ATGGCAGACGATGATCCCTAC	TGTTGACAGTGGTATTTCTGGTG
*TLR4 (Mouse)*	ATGGCATGGCTTACACCACC	GAGGCCAATTTTGTCTCCACA
*Myd88 (Mouse)*	TCATGTTCTCCATACCCTTGGT	AAACTGCGAGTGGGGTCAG
*NF-κB p65 (Mouse)*	GGAAGGATGTCTCCACACCA	AGGAACTATGGATACTGCGGTCTG
*ASC (Mouse)*	CAGCACAGGCAAGCACTCATT	TCATCTTGTCTTGGCTGGTGG
*Caspase-1 (Mouse)*	GGCTGACAAGATCCTGAGGG	TAGGTCCCGTGCCTTGTCC
*β-actin (Mouse)*	GTGACGTTGACATCCGTAAAGA	GCCGGACTCATCGTACTCC
TNF-α (human)	GAGGCCAAGCCCTGGTATG	CGGGCCGATTGATCTCAGC
IL-1β (human)	ATGATGGCTTATTACAGTGGCAA	GTCGGAGATTCGTAGCTGGA
IL-6 (human)	ACTCACCTCTTCAGAACGAATTG	CCATCTTTGGAAGGTTCAGGTTG
β-actin (human)	CATGTACGTTGCTATCCAGGC	CTCCTTAATGTCACGCACGAT

*TNF-α* tumor necrosis factor-α, *IL-1β* interleukin-1β, *CXCL2* C-X-C motif chemokine ligand 2, *MCP-1 (CCL2)* Chemokine ligand 2, *NF-κB* nuclear factor-κB, *TLR4* toll-like receptor-4, *MyD88* Myeloid differentiation factor 88, *NLRP3* NOD-like receptor thermal protein domain associated protein 3, *ASC* apoptosis- associated speck-like protein containing a CARD, *Caspase-1* Cysteine-aspartic acid protease 1

### Western blot assay

Total protein was extracted from the liver tissues and ImKCs using RIPA lysis buffer (P0013B; Beyotime, Shanghai, China). The protein concentration was quantified using a BCA assay kit (G2026; Servicebio, Wuhan, China). Samples were normalized by adding appropriate volumes of 5 × loading buffer (P0015; Beyotime, Shanghai, China) and RIPA lysis buffer prior to denaturation. The protein samples were separated by 8–15% SDS-PAGE and transferred to polyvinylidene fluoride (PVDF) membranes (Millipore, ISEQ00010, Germany). Membranes were blocked for 30 min with quick blocking buffer (G2052; Servicebio, Wuhan, China), followed by overnight incubation at 4 °C with the following primary antibodies: zonula occludens-1 (ZO-1) (rabbit, 1:2000, 21773-1-AP; Proteintech, Wuhan, China), E-cadherin (rabbit, 1:2000, 20874-1-AP; Proteintech, Wuhan, China), Occludin (rabbit, 1:2000, 27260-1-AP; Proteintech, Wuhan, China), TLR4 (mouse, 1:1000), MyD88 (mouse; 1:1000), IκBα (mouse, 1:2000, #66418-1-lg; Proteintech, Wuhan, China), p-IκBα (rabbit, 1:500, #11152; Signalway Antibody, USA), NF-κB p65 (mouse; 1:1000), p-NF-κB p65 (rabbit; 1:1000; #ab86299, Abcam, USA), NLRP3 (rabbit, 1:1000, #A21906; ABclonal, Wuhan, China), apoptosis- associated speck-like protein containing a CARD (ASC) (rabbit, 1:1000, #A16672; ABclonal, Wuhan, China), Cysteine-aspartic acid protease 1 (Caspase-1) (rabbit, 1:1000, #A0964; ABclonal, Wuhan, China), Cleaved Caspase-1 (rabbit, 1:1000, #89332; CST, USA); IL-1β (rabbit, 1:1000, #31202; CST, USA), β-actin (rabbit, 1:3000, 81115-1-RR; Proteintech, Wuhan, China). The membranes were incubated for 1 h at room temperature with species-matched horseradish peroxidase (HRP)-conjugated secondary antibodies (anti-mouse/-rabbit IgG, 1:4000, SA00001-1, SA00001-2; Proteintech, Wuhan, China). Protein bands were visualized using an enhanced chemiluminescence detection system (Bio-Rad, Hercules, California, USA), following the application of ECL substrate (P0018, Beyotime, Shanghai, China). Band intensity was quantified using the ImageJ software and normalized to β-actin.

### Transfection of siRNA

siTLR4 and si-NC were synthesized by Biomics (Jiangsu, China). Sequences: siTLR4 sense 5′-GGAUCUUUCUAAAUGUCAAUUTT-3′, antisense 5′-AAUUGACAUUUAGAAAGAUCCTT-3′; si-NC sense 5′-UUCUCCGAACGUGUCACGUdTdT-3′, antisense 5′-ACGUGACACGUUCGGAGAAdTdT-3′. Transfection used Lipofectamine 2000 (Invitrogen) refer to manufacturer's instructions. Briefly, siTLR4 and Lipofectamine were diluted in Opti‑MEM, mixed, added to ImKCs for 6 h. Medium was then replaced with DMEM/5% FBS (antibiotic‑free) for 18 h. ImKCs were then treated with LPS and/or DCHD-DS for 24 h. si‑NC served as control. Knockdown was confirmed by qRT‑PCR [40].

### Statistical analysis

The animal experiments employed 5–6 independent biological replicates per group, while the cell experiments were performed with 3–6 independent biological replicates. Statistical analyses were performed, and figures were generated using GraphPad Prism 9.5.0 (GraphPad Software, San Diego, CA, USA). Experimental data are expressed as mean ± standard deviation (SD). Differences among multiple groups were evaluated using one-way analysis of variance (ANOVA) followed by Fisher's least significant difference (LSD) post-hoc test. Comparisons between two groups were conducted using either Student's t-test or the Wilcoxon rank-sum test, as appropriate. Survival differences were analyzed using Kaplan–Meier curves, and statistical significance was assessed using the log-rank test. Correlations between parameters were examined using Pearson’s correlation analysis. Statistical significance was set at *p*-value < 0.05.

## Results

### DCHD improves CLP-induced septic mice liver injury

**Fig. 1:**
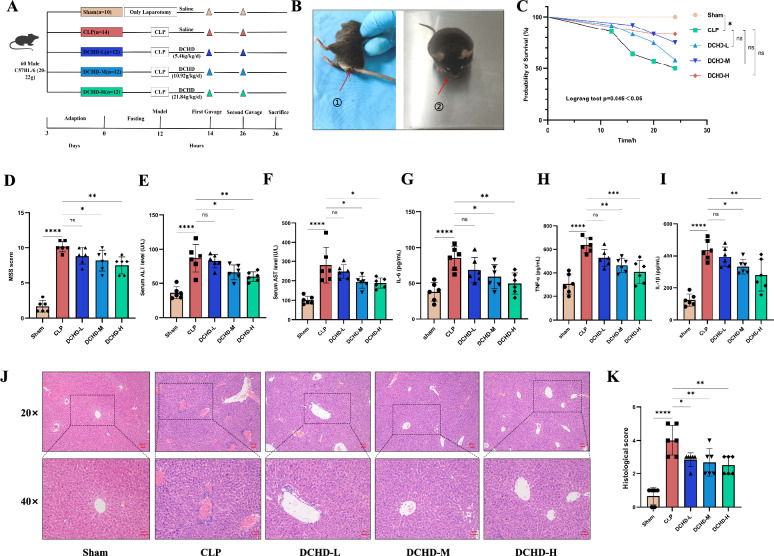
DCHD improves liver injury in CLP-induced septic mice. **A** The design of animal experiment. **B** Representative images of CLP group mice, indicated by red arrows: ① purulent exudate at the anal; ② purulent exudate at the ocular. **C** 24 h Survival curve. **D** Mss score (*n* = 6). **E**, **F** Serum levels of ALT and AST in mice (*n* = 6). **G–I** The inflammatory cytokine levels of IL-6, TNF-α, and IL-1β in serum (*n* = 6). **J** Pathological changes of liver tissues under H&E staining (scale bar = 50 μm). **K** Histological scores of liver tissues (*n* = 6)

To investigate the therapeutic efficacy of DCHD against SLI, a murine model was established via CLP, followed by interventional administration of either DCHD or saline (Fig. 1A). As shown in Fig. 1B, CLP mice exhibited characteristic features of sepsis, including lethargy, purulent ocular discharge, and perianal soiling. Moreover, Kaplan–Meier analysis showed that mice in the CLP group exhibited a 24-h mortality rate of approximately 50%, with a statistically significant difference in survival curves between the groups (log-rank test *p* = 0.045 < 0.05) (Fig. 1C). Compared to the sham group, the CLP group exhibited a significant increase in MSS, whereas DCHD treatment markedly reduced MSS in a dose-dependent manner (Fig. 1D).

To assess hepatic injury, we measured serum biomarkers and performed histopathological analysis. We found that DCHD significantly prevented CLP-induced liver injury and markedly reduced AST and ALT levels (Fig. 1E and F). Consistent with this, histological assessment showed that DCHD alleviated the marked inflammatory cell infiltration in the CLP model group in a dose-dependent manner (Fig. 1J and K). Additionally, the administration of DCHD after CLP induction dose-dependently reduced the levels of pro-inflammatory cytokines, including TNF-α, IL-6 and IL-1βin the peripheral blood of mice (Fig. 1G–I). In summary, these results confirm the successful establishment of an SLI mouse model induced by CLP. Importantly, DCHD treatment effectively alleviated liver injury by attenuating the systemic inflammation.

**Fig. 2:**
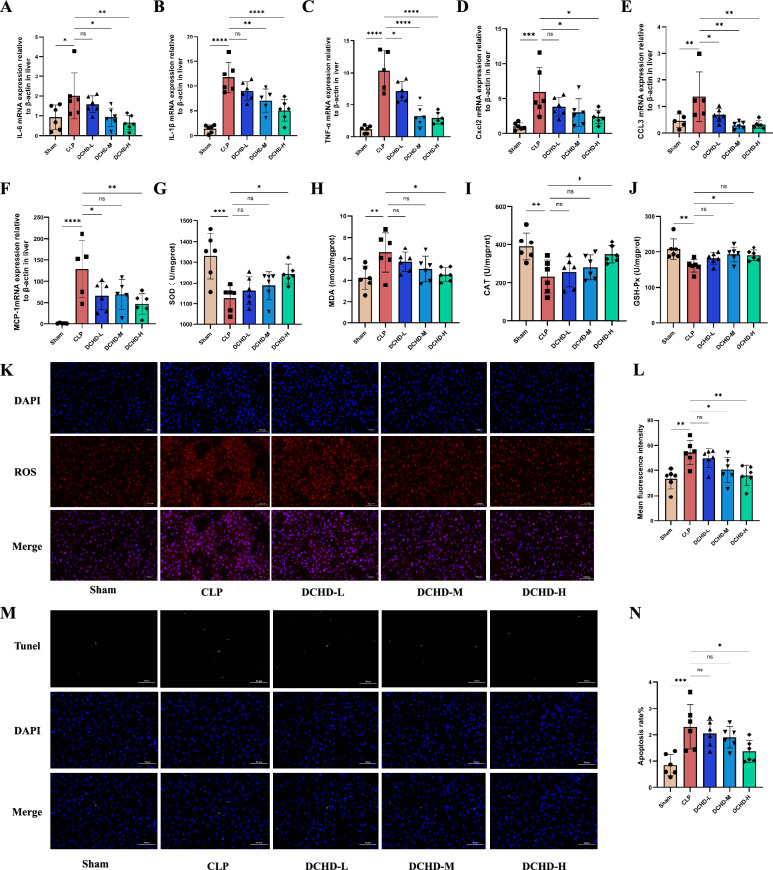
DCHD alleviates SLI in mice by suppressing hepatic inflammation, oxidative stress, and hepatocyte apoptosis. **A–F** The mRNA expression levels of *IL-6, IL-1β, TNF-α, CXCL2, CCL3*, and *MCP-1* in liver tissues detected using qRT-PCR (*n* = 5—6). **G–J** SOD, MDA, CAT, and GSH-Px levels in liver tissues detected using commercial assay kits (*n* = 6). **K**, **L** Detection and quantification of ROS in liver tissues were performed using cryosectioning coupled with fluorescent probe technology (*n* = 6). **M**, **N** TUNEL staining of liver tissues and quantitative analysis of the apoptotic rate (*n* = 6). Date are presented as the mean ± SD. *p < 0.05, **p < 0.01, ***p < 0.001, and ****p < 0.0001 vs. CLP group

Further evaluation was performed to determine whether the therapeutic potential of DCHD in ameliorating SLI involved inhibition of liver inflammation and oxidative stress. As anticipated, we found that the CLP group exhibited significant upregulation of *IL-6, IL-1β, TNF-α, CXCL2, CCL3*, and *MCP-1* mRNA in liver tissues compared to the sham group, while DCHD dramatically decreased their expression (Fig. 2A–F). Subsequently, we measured the levels of SOD, MDA, GSH-Px, and CAT in the mouse liver tissues. The results indicated that DCHD significantly elevated the activities of the antioxidant enzymes SOD, GSH-Px, and CAT while reducing the level of MDA, a marker of lipid peroxidation (Fig. 2G–J). The overproduction of ROS contributes to oxidative stress and the associated liver injury. The CLP group demonstrated a marked increase in ROS levels relative to the sham group, demonstrating substantial oxidative stress. Conversely, the DCHD group exhibited a pronounced reduction in ROS levels, indicating that the intervention effectively attenuated the oxidative damage (Fig. 2K and L). The TUNEL assay was used to further detect the breakdown of nuclear DNA during apoptosis, and the results demonstrated a significant increase in the percentage of TUNEL-positive (apoptotic) cells in the liver tissues of CLP-treated mice, whereas DCHD treatment significantly attenuated apoptosis (Fig. 2M and N). In summary, these observations demonstrate that DCHD confers hepatoprotective effects in septic mice by inhibiting systemic inflammation and oxidative stress, which, in turn, limits hepatocyte apoptosis.

### UPLC-Q-TOF–MS analysis

**Fig. 3:**
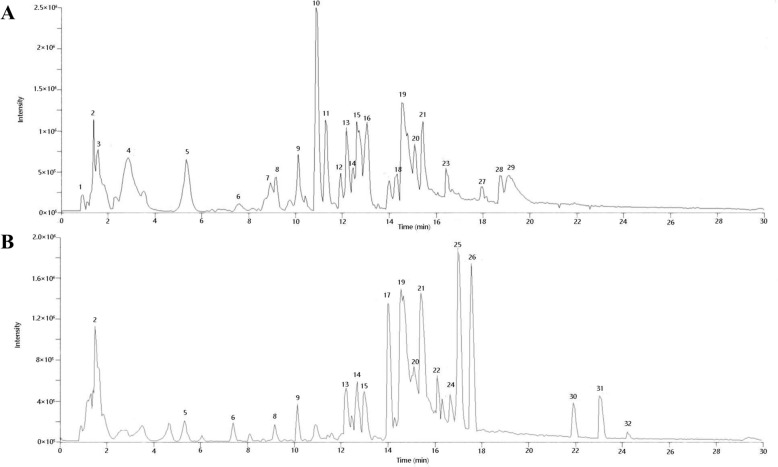
The total ion chromatograms of DCHD obtained using HPLC-Q-TOF–MS. **A** The total ions current under positive ion mode. **B** The total ions current under negative ion mode

To profile the major chemical components of DCHD, HPLC-Q-TOF–MS analysis was conducted in positive and negative ion modes (Fig. 3A and B). We conducted a quality evaluation of the DCHD samples, and the results demonstrated good batch-to-batch consistency, indicating that the data are stable and reliable. A total of 32 major components that exhibited high response intensities were identified. The detailed compound information is presented in Supplementary Material 2, including the identification names of monomeric components, molecular formula, m/z, RT, reference ion, and 2D structure. Baicalin, lobeline, and kaempferol identified in DCHD have been confirmed to be effective against sepsis-related organ failure [41–43].

### Network pharmacology analysis

**Fig. 4:**
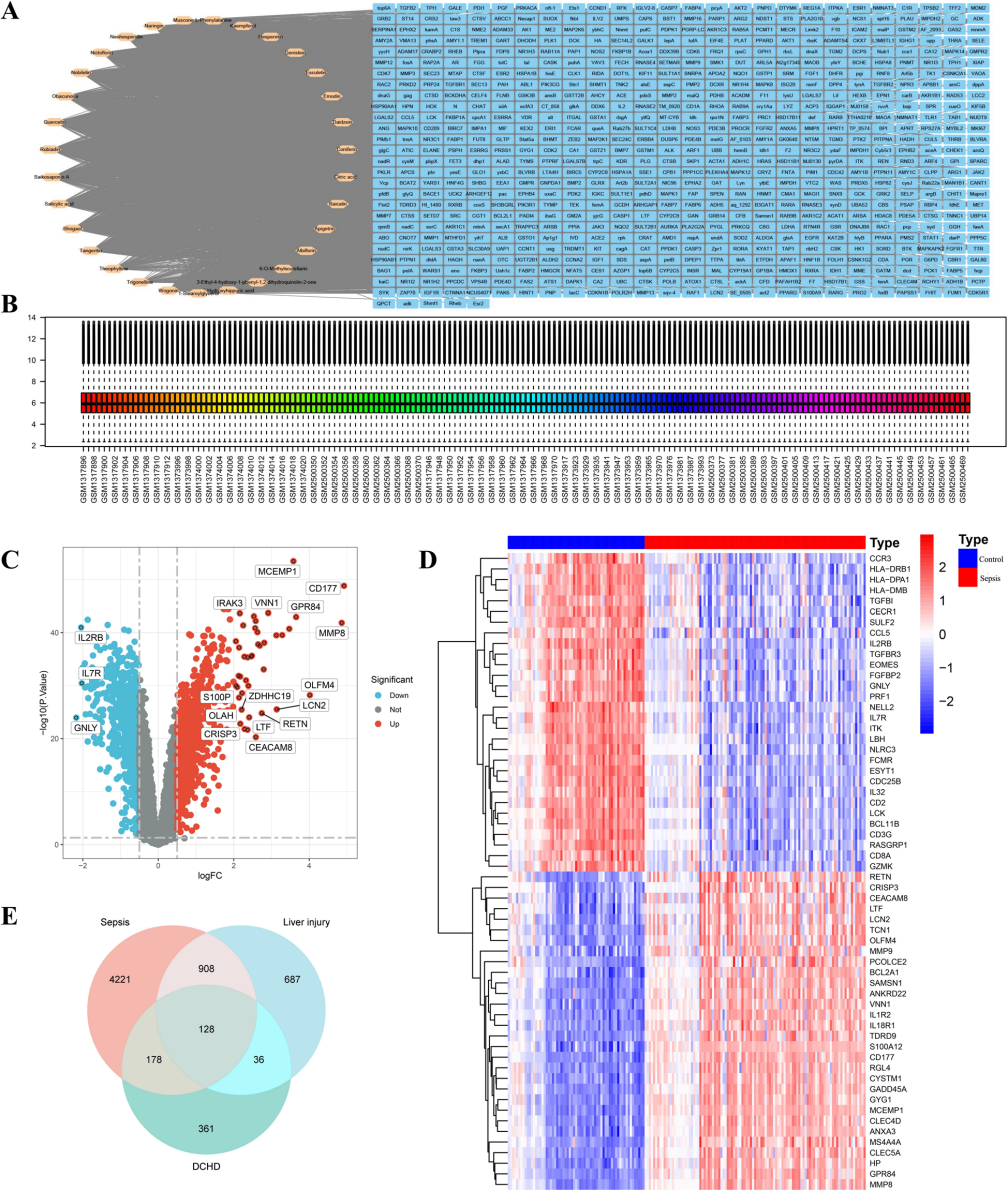
Network pharmacology analysis of DCHD in the treatment of SLI. **A** The components-targets network of DCHD. In this network, the orange hexagons represent the core components of DCHD, while the blue rectangles represent the respective targets. **B** Distribution of normalized raw data across samples. **C** Volcano plot depicting sepsis-related DEGs. Up-regulated, down-regulated, and non-significant genes are denoted in red, green, and grey, respectively. **D** Heatmap of the sepsis-related DEGs. Expression levels are color-coded, with blue indicating low expression and red indicating high expression. **E** Venn diagram showing the intersection among DCHD, sepsis, and liver injury

A total of 703 prospective targets of DCHD were identified through an integrated analysis of UPLC-Q-TOF–MS data and the SwissTargetPrediction platform, and a “Components-targets network diagram” was constructed by Cytoscape 3.7.0 (Fig. 4A). Genistein, Wogonin, and Kaempferol exhibited the highest degree of connectivity within the network, suggesting their potential roles as the primary active components of DCHD against SLI. What’s more, the microarray datasets GSE54514, GSE57065, and GSE95233 were obtained from the GEO database, comprising 65 samples from control subjects and 105 samples from the sepsis group. Normalization of these samples was performed using a box plot, as illustrated in Fig. 4B. Using a significance threshold of *p*-value < 0.05 and |log_2_FC|≥ 0.5, we identified 1595 DEGs, comprising 790 upregulated and 805 downregulated genes. Differential expression analysis was graphically represented in a heat map and volcano plot (Fig. 4C and D). Additionally, 4321 targets associated with sepsis and 1759 targets linked with liver injury were identified from the GeneCard database. Finally, the targets related to DCHD, sepsis, and liver injury were mapped using the Jvenn platform, which identified 128 overlapping targets (Fig. 4E).

**Fig. 5:**
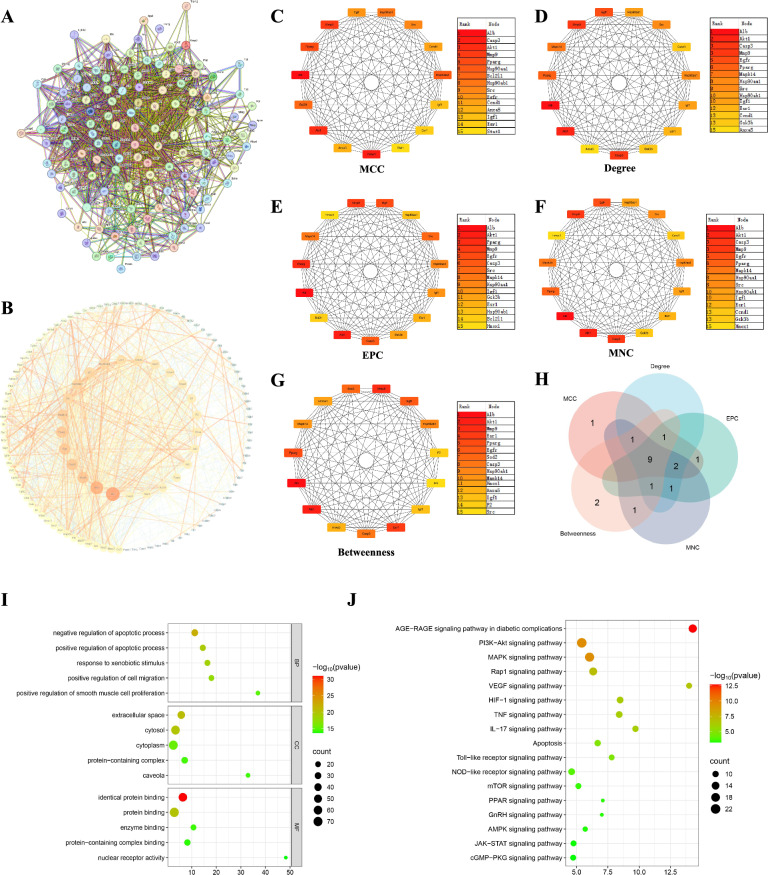
Identification of core target genes and enrichment Analysis. **A**, **B** The PPI network was constructed using the STRING database and visualized with Cytoscape 3.7.0. In the PPI network, targets of higher significance are represented by nodes that are larger in size, darker in color, and connected by thicker lines. **C–G** The top 15 targets identified respectively by the MCC, Degree, EPC, MNC, and Betweenness Centrality algorithms in the CytoHubba plugin. **H** The core target genes were visualized using a flower Venn diagram, which were identified by intersecting the top 15 genes from each of the five algorithms in the CytoHubba plugin: MCC, Degree, EPC, MNC, and Betweenness Centrality. **I** Bubble plot visualization of the top five enriched terms across all three GO categories (BP, CC, MF). Larger bubble sizes denote a greater number of enriched genes, while a deeper color indicates a higher level of enrichment significance. **J** Bubble plot of KEGG pathway enrichment

Based on a set of 128 overlapping targets, we imported them into the STRING database and subsequently downloaded the corresponding “TSV” file, and a PPI network was then constructed using Cytoscape 3.7.0. (Fig. 5A and B). Core targets were identified by integrating the top 15 targets derived from MCC, Degree, EPC, MNC, and Betweenness Centrality algorithms within the CytoHubba plugin. These core targets included ALB, CASP3, AKT1, MMP9, PPARG, HSP90AB1, SRC, EGFR, and IGF1 (Fig. 5C–H). GO analysis identified 1472 significantly enriched terms, comprising 598 BP terms, 66 CC terms, and 238 MF terms. The top five most significantly enriched terms from each category are presented in a bubble chart (Fig. 5I). Among the BP terms, the ten most highly enriched were primarily associated with negative regulation of the apoptotic process, positive regulation of the apoptotic process, response to xenobiotic stimulus, positive regulation of cell migration, and positive regulation of smooth muscle cell proliferation. For MF, the top terms were related to functions, such as identical protein binding, protein binding, enzyme binding, protein-containing complex binding, and nuclear receptor activity. The leading CC terms included extracellular space, cytosol, cytoplasm, protein-containing complex, and caveola. KEGG pathway analysis demonstrated that the targets of DCHD for SLI treatment were significantly enriched in pathways associated with inflammatory reaction-related pathways (e.g., TNF, IL-17, and toll-like receptor signaling pathways), cell proliferation and metabolism pathways (e.g., PI3K-Akt, MAPK, and Rap1 signaling pathways), and angiogenesis and hypoxia response pathways (e.g., VEGF, HIF-1, and apoptosis pathways) (Fig. 5J).

### Molecular docking

**Fig. 6:**
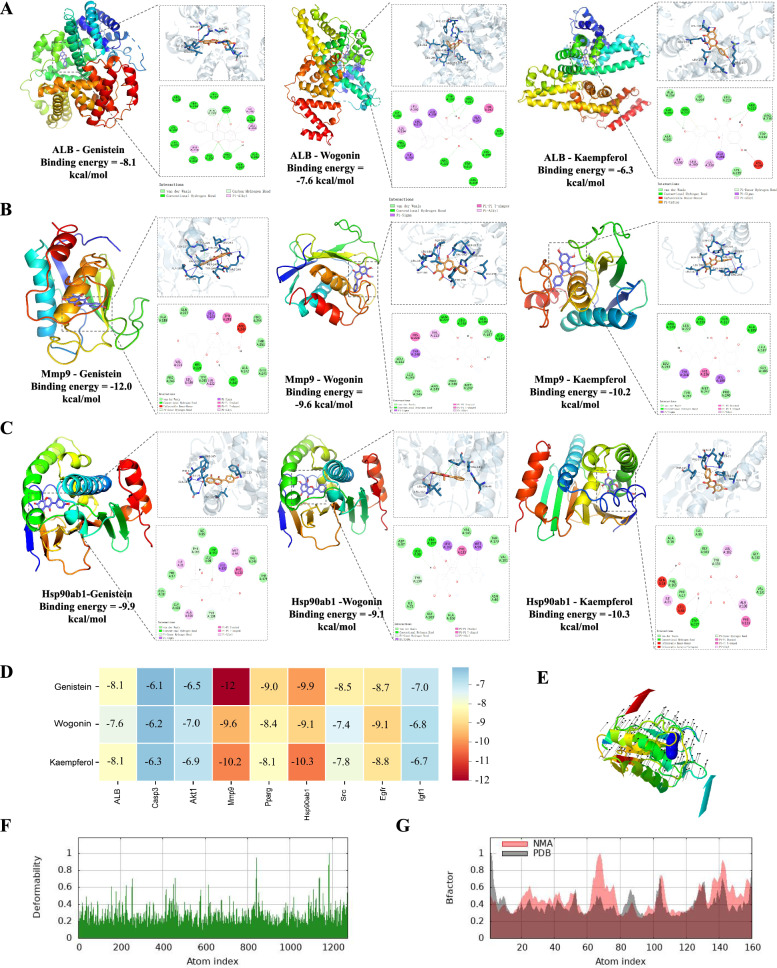
Molecular docking and molecular dynamics simulation. **A** Molecular docking of ALB with Genistein, Wogonin, and Kaempferol. **B** Molecular docking of Mmp9 with Genistein, Wogonin, and Kaempferol. **C** Molecular docking of Hsp90ab1 with Genistein, Wogonin, and Kaempferol. **D** Heatmap of the binding energies between the three DCHD core components and nine core target genes. **E** Molecular motion visualization. The small and large arrows indicate the direction of individual amino acid movement and the overall motion trend of the protein, respectively. **F** Visualization of the ligand–protein binding deformability. **G** B-factor profiles comparing atomic thermal vibration amplitudes derived from Normal Mode Analysis (NMA) and the Protein Data Bank (PDB)

Computational docking studies were conducted to assess the intermolecular binding of three potentially bioactive components and nine primary candidate targets. Ligand-receptor binding affinities are generally considered strong, good, or certain when the binding energies are below − 7.0, − 5.0, and − 4.0 kcal/mol, respectively [28]. This inverse correlation between binding energy and affinity indicates that lower binding energies are associated with higher ligand-receptor binding strength and greater conformational stability. The results of molecular docking revealed that the binding energies of the receptor—ligand interactions were consistently < − 5.0, indicating good binding and robust structural stability between the receptors and ligands. Notably, Genistein, Wogonin, and Kaempferol exhibited lower binding energies for ALB, MMP9, and HSP90AB1 (Fig. 6A–D). These findings suggest that the three characteristic components of DCHD form stable interactions with the core targets ALB, MMP9, and HSP90AB1, probably exerting considerable influence on SLI treatment. Molecular dynamics simulations of the binding between Genistein and MMP9 revealed a moderate peak in flexibility, indicating a favorable docking outcome. Furthermore, the post-docking B-factor fluctuations remained within an acceptable range and did not disrupt the natural motion modes of the protein (Fig. 6E–G).

### Effect of DCHD on intestinal barrier integrity in CLP-induced septic mice

**Fig. 7:**
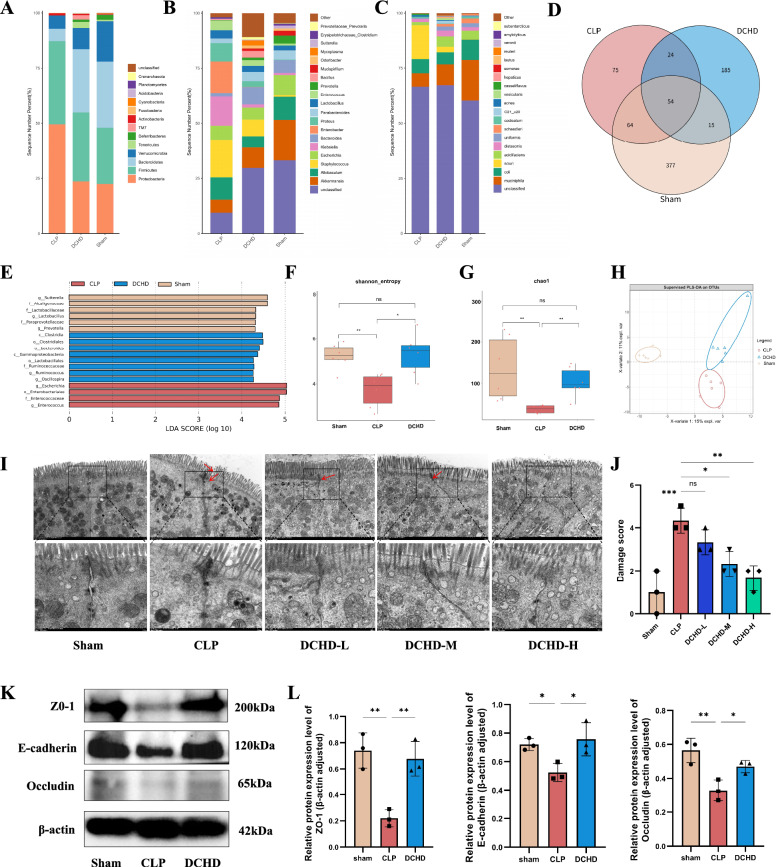
DCHD ameliorates the disruption of intestinal barrier function in mice with CLP-induced SLI. **A–C** Bar chart of relative distribution of each group at the phylum, genus, and species levels. The horizontal axis represents the group name, and the vertical axis indicates the ratio of the number of sequences annotated to the levels of the phylum, genus, and species to the total annotated data, respectively. The color order of the bar chart from top to bottom corresponds to the color order of the legend on the right. **D** Venn diagram of the number of OTUs between different subgroups. **E** LEfSe analysis LDA bar chart. Each horizontal bar represents a species, and the length of the bar corresponds to the LDA value, with higher LDA values indicating greater variation. The color of the bar corresponds to which subgroup of characteristic microorganisms the species is a part of, and characteristic microorganisms indicate relatively high abundance in the corresponding subgroup. **F**, **G** Diversity of intestinal flora based on Shannon and Chao1 indices (*n* = 6). **H** PLS-DA analysis. Each point of the PLS-DA coordinate plot represents a sample, points with the same color belong to the same grouping, and points of the same grouping are marked with ellipses. If the samples belonging to the same grouping are closer to each other, and at the same time the distance between the points of different groupings is farther, it indicates that the classification model is more effective. **I** The ultrastructure of the colonic epithelium of mice under TEM, the red arrows represent TJs (Scale bar: 2 μm and 500 nm). **J** The damage score (*n* = 3). **K**, **L** Western blot bands and relative protein expression levels of ZO-1, E-cadherin, and Occludin in colon tissues (*n* = 3). Date are presented as the mean ± SD. *p < 0.05, **p < 0.01 vs. CLP group

The gut microbiota is essential for intestinal barrier integrity and modulates liver function via the gut—liver axis [34]. Therefore, we analyzed the gut microbiota structure modified by DCHD using 16S rRNA sequencing. As illustrated in Fig. 7A–C, CLP mice exhibited increased *Proteobacteria* and decreased *Bacteroidetes* and *Verrucomicrobia* at the phylum level compared with the sham group. Treatment with DCHD reversed these changes. *Firmicutes* remained stable across all groups. At the genus level, CLP mice showed reduced *Akkermansia* and elevated opportunistic genera such as *Staphylococcus*, *Klebsiella*, *Enterobacter*, and *Proteus*. DCHD restored the genus-level composition to a profile similar to that in the sham-operated group. Species-level analysis revealed a decrease in *muciniphila* levels and an increase in *Staphylococcus sciuri* levels in CLP mice, which normalized after DCHD treatment. In summary, CLP-induced SLI leads to gut dysbiosis, characterized by reduced obligate anaerobes, increased facultative anaerobes, and proliferation of pathobionts. DCHD intervention effectively restored microbial homeostasis, resembling the sham group.

Venn diagram analysis of the microbial features across multiple groups revealed 54 shared operational taxonomic units (OTUs) among the three cohorts. The sham group exhibited the highest number of unique OTUs, whereas the CLP group exhibited the lowest number of unique OTUs (Fig. 7D). To identify the key differential bacterial genera, LEfSe analysis was performed. Notably, the sham group was characterized by *Sutterella*, *Alcaligenaceae*, and members of the genera *Lactobacillus* and *Prevotella*. In contrast, the CLP group exhibited enriched features primarily within the order *Enterobacteriales*, including the family/genus *Enterococcaceae* and the genus *Escherichia*. The DCHD group showed a predominant representation of taxa spanning several phylogenetic levels, including the class *Clostridia*, order *bacteroides*, class γ-proteobacteria, order *Lactobacillales*, family/genus *Ruminococcus* and genus *Oscillospira* (Fig. 7E). Distinct microbial signatures were observed between the CLP and sham groups. The sham group was predominantly characterized by beneficial bacteria, such as *Lactobacillus*, whereas the CLP group was primarily characterized by opportunistic pathogens, including *Enterobacter* and *Enterococcus*. In contrast, the DCHD group exhibited a higher prevalence of taxa, including *β-proteobacteria* and *Lactobacillales*. We assessed the α-diversity of species within each group using the Shannon and Chao1 indices, with elevated index values indicating greater microbial diversity. The results indicated that the CLP group exhibited significant dysbiosis, characterized by an altered gut microbiota structure, accompanied by a reduction in diversity. Conversely, DCHD treatment increased the diversity of gut microbiota in septic mice, resulting in a microbial composition that more closely resembled that of the sham group (Fig. 7F–H).

TEM examination of colonic tissues revealed well-arranged intestinal epithelial cells with intact tight junctions (TJs) and normal microvilli in the sham controls, whereas the CLP group exhibited disrupted TJs and microvilli, markedly impaired gut barrier integrity, increased intestinal permeability, and significantly elevated ultrastructural damage scores (*p* < 0.001). The DCHD group, particularly at a high dose, showed comparatively preserved intestinal architecture and a significant reduction in colonic ultrastructural injury (*p* < 0.01) (Fig. 7I and J). Furthermore, we evaluated the impact of DCHD on the intestinal mucosal barrier in mice by measuring the expression of ZO-1, Occludin, and E-cadherin using western blotting. Compared to the sham group, the protein levels of ZO-1, Occludin, and E-cadherin were significantly reduced in the CLP group, which was reversed following DCHD treatment (Fig. 7K and L). The sequencing results indicated that CLP-induced SLI compromised the intestinal barrier integrity and increased intestinal permeability in mice. Treatment with DCHD ameliorates these pathological alterations, thereby restoring mucosal barrier function and modulating the gut microbiota composition.

### Gut-derived LPS translocates to the liver and activates Kupffer cells (KCs)

**Fig. 8:**
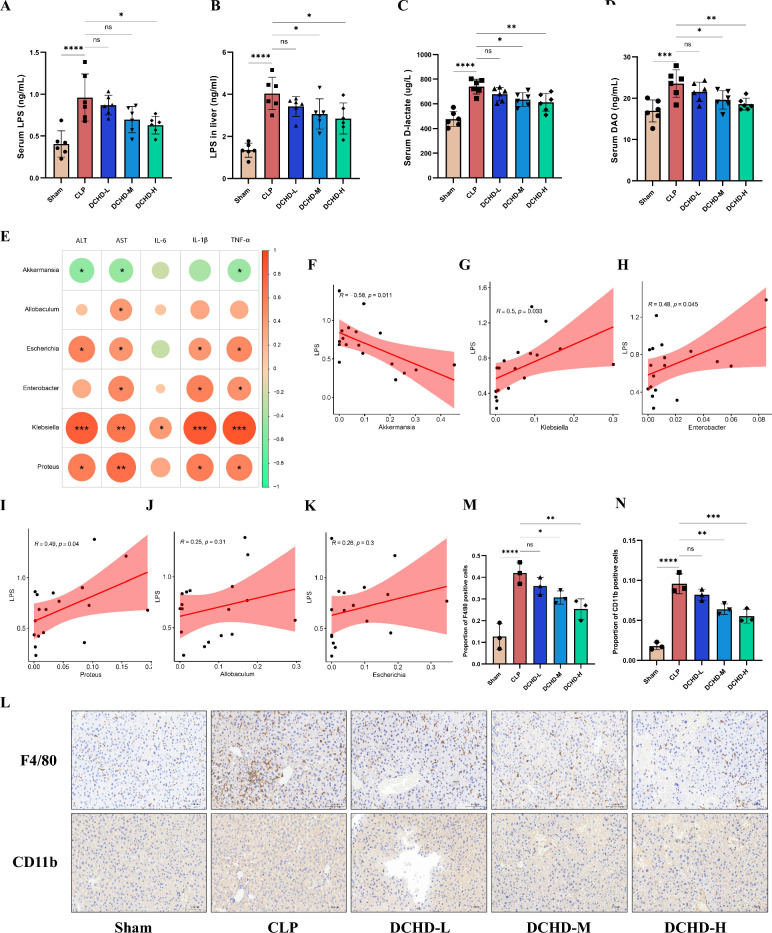
LPS is the key mediator of crosstalk between the gut and liver. **A** Serum levels of LPS (*n* = 6). **B** Liver levels of LPS (*n* = 6). **C**, **D** The serum levels of D-lactate and DAO (*n* = 6). **E** Correlation heatmap of differential gut microbiota and liver injury markers. **F** Correlation between *Akkermansia* and serum LPS levels. **G** Correlation between *Klebsiella* and serum LPS levels. **H** Correlation between *Enterobacter* and serum LPS levels. **I** Correlation between *Proteus* and serum LPS levels. **J** Correlation between *Allobaculum* and serum LPS levels. **K** Correlation between *Escherichia* and serum LPS levels. **L–N** IHC detection of F4/80 and CD11b proteins in liver sections with quantifications (*n* = 3). Date are presented as the mean ± SD. *p < 0.05, **p < 0.01, ***p < 0.001, and ****p < 0.0001 vs. CLP group

LPS, an integral structural component of the outer membrane in gram-negative bacteria, serves not only as a pivotal biomarker of intestinal barrier integrity but also as a central mediator in gut-liver axis communication. To this end, we quantified LPS levels in both serum samples and liver tissues to elucidate the interconnection between the gut and liver. Quantitative analysis revealed a significant accumulation of LPS in the serum and liver tissues of CLP-induced mice relative to the sham group. Conversely, administration of DCHD markedly reduced these levels (Fig. 8A and B). The serum levels of D-lactate and DAO, markers of bacterial translocation, and intestinal permeability were also quantified. Compared to the sham group, CLP-induced septic mice exhibited a significant increase in systemic markers of intestinal permeability (D-lactate and DAO), which was subsequently mitigated by DCHD intervention (Fig. 8C and D). We performed Spearman correlation analysis to preliminarily explore the association between key differential gut microbiota and the therapeutic efficacy of DCHD. The results revealed that *Akkermansia* exhibited a significant negative correlation with ALT, AST, and TNF-α levels (*p* < 0.05). In contrast, *Escherichia*, *Enterobacter*, *Klebsiella*, and *Proteus* showed positive correlations with ALT, AST, IL-1β, and TNF-α, among which *Klebsiella* demonstrated the strongest correlation with these markers (*p* < 0.01) (Fig. 8E). To investigate the association between elevated serum LPS levels and intestinal dysbiosis, we analyzed the correlation between LPS levels and relative abundance of abundant bacterial genera. The results revealed a significant negative correlation between *Akkermansia* and serum LPS levels (*R* = −0.58, *p* < 0.05) (Fig. 8F). In contrast, no significant correlation was observed between *Allobaculum* and *Escherichia* (Fig. 8J and K). Notably, *Klebsiella*, *Enterobacter*, and *Proteus* were positive correlated with serum LPS levels (*R* = 0.50, 0.48, and 0.49, respectively; *p* < 0.05) (Fig. 8G–I). Collectively, these findings indicate that increased serum LPS levels are closely linked to the structural disruption of the gut microbiota and overgrowth of gram-negative bacilli. Specifically, *Klebsiella*, *Proteus*, and *Enterobacter* may contribute to LPS production, which subsequently translocates into the bloodstream through an impaired intestinal barrier, directly influencing host immune and metabolic regulation.

As resident macrophages of the liver, KCs constitute a critical sentinel defence against the translocation of intestinal-derived pathogens. They orchestrate the innate immune response by proficiently recognizing and phagocytosing circulating microbes and their products, notably LPS, via pattern recognition receptors such as TLR4. This recognition triggers the robust secretion of a cascade of pro-inflammatory mediators, including cytokines (e.g., TNF-α, IL-1β, and IL-6) and chemokines, thereby initiating a potent anti-infectious immune response that is indispensable for containing systemic infection [44]. To directly visualize and quantify the hepatic macrophage response in our septic model, we employed IHC for two canonical markers: F4/80, a highly specific antigen for murine mature tissue-resident macrophages, and CD11b, an integrin that is abundantly expressed in both activated and recruited monocytes [45]. The expression of the macrophage markers F4/80 and CD11b was markedly upregulated in the liver tissues of CLP-induced septic mice (*p* < 0.0001), and this upregulation was effectively suppressed by DCHD treatment (*p* < 0.01) (Fig. 8K–M). Taken together, these data suggest that gut-derived LPS may trigger a cascade of hepatic inflammatory immune responses via KCs activation, which can ameliorate the dysregulated liver immune microenvironment in mice with sepsis.

### Effect of DCHD on the hepatic transcriptional profile in mice with CLP-induced SLI

**Fig. 9:**
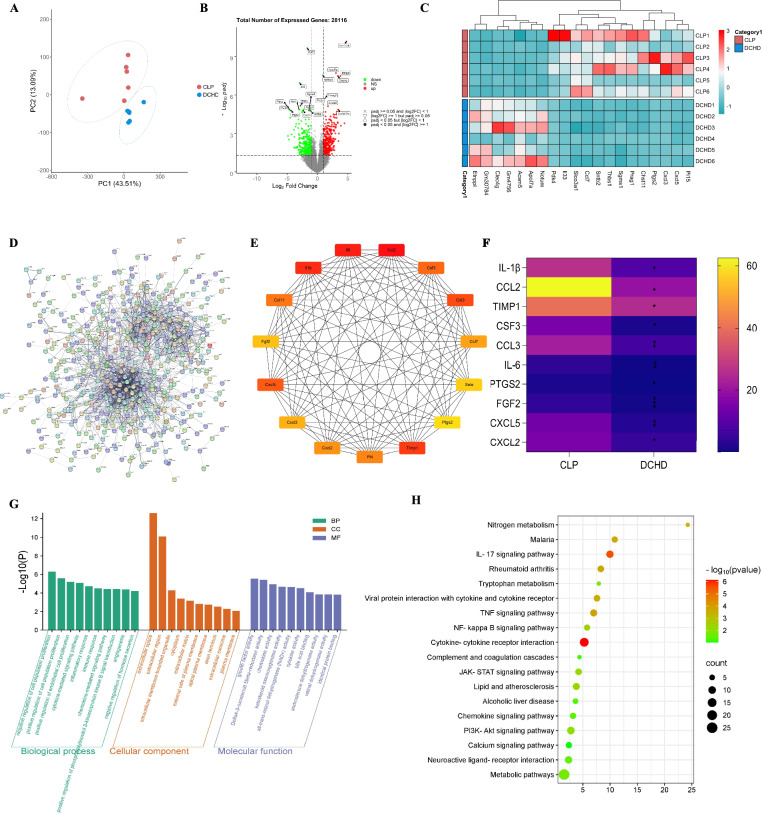
DCHD regulates liver transcriptomic profile in mice with CLP-induced SLI. **A** The result of PCA. **B** The volcano map of DEGs. **C** The cluster heatmap of genes with the most significant differences. **D** PPI network of DEGs. **E** The network diagram of 15 candidate genes. **F** The expression levels of core genes (IL-1β, CCL2, TIMP1, CSF3, CCL3, IL-6, PTGS2, FGF2, CXCL5 and CXCL2). **G** GO enrichment analysis. **H** Bubble chart of KEGG pathway analysis. Date are presented as the mean ± SD. *p < 0.05, **p < 0.01, and ***p < 0.001 vs. CLP group

We used comprehensive RNA sequencing to investigate hepatic transcription. Intriguingly, Principal component analysis (PCA) presented pronounced disparities between the DCHD and CLP groups, whereas samples within each group exhibited tight clustering, indicating high intra-group consistency (Fig. 9A). Using thresholds of |log_2_(fold change)|> 1 and an adjusted p-value (padj) < 0.05, we identified 656 DEGs, comprising 354 upregulated and 302 downregulated genes (Supplementary Material 3). The overall distribution of DEGs and expression patterns of key genes were visually summarized using volcano plots and heat maps, respectively (Fig. 9B and C). Based on PPI network construction, topological assessment, and module analysis, the top 15 candidate genes were selected for further evaluation (Fig. 9D–F; Table 2). Among the BP terms, cell population proliferation, cytokine-mediated signaling pathways, inflammatory responses, and immune responses were mainly involved. For MF, the top terms were growth factor activity, Delta4-3-oxosteroid 5beta-reductase activity, and chemokine activity. CC primarily comprised the extracellular space, extracellular region, intracellular membrane-bound organelles, cytoplasm, and extracellular matrix (Fig. 9G). Furthermore, KEGG enrichment analysis highlighted several pivotal signaling pathways, including inflammatory response, chemokine-mediated signaling, TNF signaling, IL-17 signaling, PI3K-Akt signaling, JAK-STAT signaling, and NF-κB signaling, which are likely central to the pathophysiology of DCHD against SLI (Fig. 9H). Collectively, these findings suggest that DCHD ameliorates hepatic inflammation by suppressing the production of pro-inflammatory mediators via modulation of the NF-κB signaling pathway.

**Table 2:** The core genes identified by the PPI network and topology and module analysis

No.	Gene	Protein names	Betweenness Centrality	Closeness Centrality	Degree
1	Il-6	Interleukin-6	0.24109033	0.69852941	66
2	Il-1β	Interleukin-1β	0.14374053	0.65517241	60
3	Ccl2	C-C motif chemokine 2	0.08912526	0.625	56
4	Timp1	Tissue inhibitor of metal protease 1	0.04441489	0.54285714	37
5	Ccl3	C-C motif chemokine 3	0.02200713	0.53370787	36
6	Fgf2	Fibroblast growth factor 2	0.06204792	0.53370787	36
7	Ptgs2	Prostaglandin G/H synthase 2	0.02010354	0.52777778	36
8	Csf3	Colony stimulating factor 3	0.01773561	0.52486188	34
9	Hif1a	Hypoxia-inducible factor-1α	0.029629	0.53072626	32
10	Cxcl5	C-X-C motif chemokine 5	0.00561303	0.4822335	27
11	Cxcl2	C-X-C motif chemokine 2	0.00620943	0.47979798	25
12	Ccl11	C-C motif chemokine ligand 11	0.00383437	0.47263682	23
13	Pf4	Platelet factor 4	0.01006971	0.5	23
14	Thbs1	Thrombospondin-1	0.00885058	0.47979798	23
15	Edn1	Endothelin 1	0.00370968	0.47029703	22

*IL-6* interleukin-6, *IL-1β* interleukin-1β, *CCL2* Chemokine ligand 2, *TIMP1* Tissue Inhibitor of Metalloproteinases 1, *FGF2* Fibroblast growth factor 2, *Ptgs2* Prostaglandin-Endoperoxide Synthase 2, *Csf3* colony stimulating factor 3, *Hif1a* Hypoxia Inducible Factor 1a, *Cxcl5* C-X-C motif chemokine ligand 5, *Pf4* Platlet factor 4, *Thbs1* Thrombospondin-1, *Edn1* Endothelin-1

### DCHD ameliorates sepsis-induced liver injury by regulating TLR4/NF-KB/NLRP3/Caspase-1 pathway in vivo

**Fig. 10:**
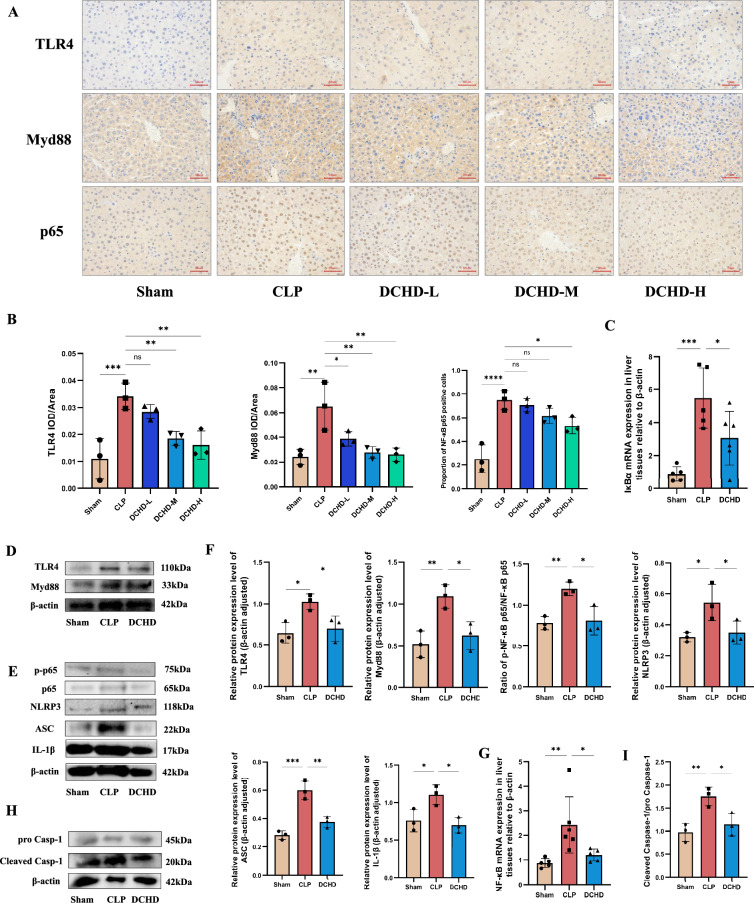
DCHD ameliorates sepsis-induced liver inflammatory injury by regulating TLR4/NF-κB/NLRP3/Caspase-1 pathway in CLP-induced septic mice. **A-B** IHC detection of TLR4, Myd88, and NF-κB p65 proteins in liver sections with quantifications (*n* = 3). **C** The mRNA expression level of *IκBα* in liver tissues (*n* = 5—6). **D–F** Representative Western blot bands and the quantitative analysis results of TLR4, Myd88, p-NF-κB p65 NLRP3, ASC, and IL-1β in the liver tissues (β-actin adjusted) (*n* = 3). **G** Relative mRNA expression level of *NF-κB* in liver tissues (*n* = 5—6). **H**, **I** Representative Western blot bands and the quantitative analysis results of Cleaved Caspase-1 in the liver tissues (β-actin adjusted) (*n* = 3). Date are presented as the mean ± SD. *p < 0.05, **p < 0.01, and ***p < 0.001 vs. CLP group

TLR4 is a transmembrane signaling receptor that is critical for detecting pathogen-associated molecular patterns (PAMPs) such as LPS [46]. Integrated network pharmacology and transcriptomic analyses have revealed that NF-κB is one of the central pathways mediating the protective effects of DCHD against SLI. Therefore, we propose that gut dysbiosis initiates hepatic NF-κB signaling via the LPS-TLR4 axis, subsequently activating the NLRP3/Caspase-1 inflammasome and exacerbating the inflammatory microenvironment in the liver, a process that may be modulated by DCHD. Initially, we assessed the protein expression levels of TLR4, Myd88, and NF-κB p65 in the liver tissues via IHC. The results revealed significantly elevated expression of TLR4, Myd88, and NF-κB p65 in the CLP group compared to the sham group (*p* < 0.01). Following treatment with DCHD, the expression of these proteins was markedly reduced in a concentration-dependent manner, with the most pronounced effect observed in the DCHD-H group (*p* < 0.05) (Fig. 10A and B). Therefore, we selected a medium dose of DCHD for further studies. Similarly, both qRT-PCR and western blot assays demonstrated that DCHD suppressed the expression of TLR4, Myd88, p-IκBα, and p-NF-κB p65 in the liver tissues (Fig. 10C–G). Furthermore, we assessed the expression of NLRP3, ASC, cleaved caspase-1, and IL-1β in liver tissues. The CLP group exhibited marked upregulation of these proteins compared with the sham group. In contrast, DCHD treatment effectively suppressed their expression, consequently abrogating the assembly of the NLRP3 inflammasome and the subsequent IL-1β-driven inflammatory response (Fig. 10E, F, H and I). These findings suggest that DCHD ameliorates sepsis-induced liver inflammatory injury by regulating the TLR4/NF-KB/NLRP3/Caspase-1 signaling pathway in CLP-induced septic mice.

### DCHD-DS mitigates LPS-induced inflammatory injury in ImKCs via inhibition of the NF-κB/NLRP3/Caspase-1 pathway

**Fig. 11:**
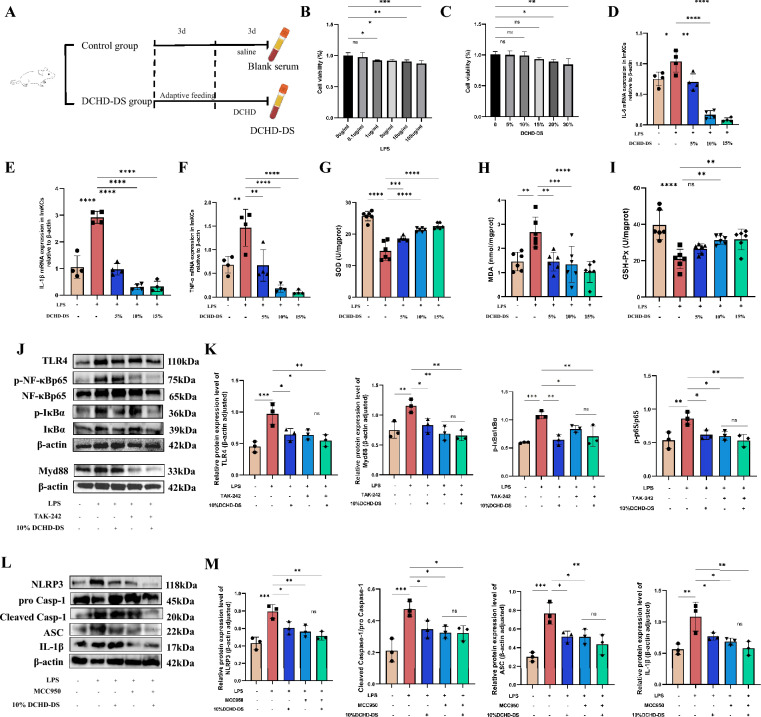
DCHD-DS alleviates LPS-induced inflammation and oxidative stress in ImKCs by suppressing the NF-κB/NLRP3/Caspase-1 signaling pathway. **A** Schematic diagram of the experimental preparation of DCHD-DS. **B** Determination of the optimal LPS concentration using the CCK-8 assay (*n* = 6). **C** Determination of the optimal DCHD-DS concentration using the CCK-8 assay (*n* = 6). **D–F** mRNA expression levels of I*IL-6, IL-1β*, and *TNF-α* in ImKCs (*n* = 4). **G–I** Relative SOD, MDA, and GSH-Px levels in ImKCs (*n* = 6). **J–M** Representative Western blot bands and the quantitative analysis results of TLR4, Myd88, p-IκBα, p-NF-κB p65, NLRP3, ASC, Cleaved Caspase-1, and IL-1β in ImKCs (β-actin adjusted) (*n* = 3). Date are presented as the mean ± SD. *p < 0.05, **p < 0.01, ***p < 0.001, and ****p < 0.0001

In vitro, the intervention was performed using the prepared DCHD-DS and rigorous quality *control* measures were implemented (Fig. 11A). In our study, ImKCs were stimulated with 1 μg/mL LPS and concurrently treated with 5%, 10%, and 15% DCHD-DS, as determined by CCK-8 assay (Fig. 11B and C). Subsequent qRT-PCR analysis revealed that LPS stimulation markedly upregulated the mRNA expression of *IL-6*, *IL-1β*, and *TNF-α* in ImKCs compared to the control group. Conversely, DCHD-DS treatment significantly attenuated this induction (Fig. 11D–F). We observed that DCHD-DS attenuated LPS-induced oxidative stress in ImKCs, as evidenced by the elevated MDA levels and reduced SOD and GSH-Px activities in the model group, whereas DCHD-DS treatment significantly decreased MDA levels and enhanced the activities of both SOD and GSH-Px (Fig. 11G–I).

To further validate whether DCHD-DS could alleviate LPS-induced inflammatory injury in ImKCs via the NF-κB/NLRP3/Caspase-1 signaling axis in vitro, we used western blot analysis to assess the protein expression levels of key components, including TLR4, MyD88, p-IκBα, and p-NF-κB p65 in ImKCs. As shown in Fig. 11J and K, compared with the control group, TLR4, MyD88, p-IκBα, and p-NF-κB p65 protein levels were markedly upregulated in the model group (*p* < 0.05). To determine whether DCHD-DS exerted its effects by targeting TLR4 or its downstream signaling pathways, we employed TAK-242 (a TLR4 inhibitor). Western blot analysis revealed that the levels of TLR4 and its downstream associated proteins were markedly downregulated in the LPS + TAK-242 group compared with those in the model group. Similarly, DCHD-DS treatment, rather than TAK-242 treatment, produced a comparable effect (*p* < 0.05). Notably, when TLR4 was inhibited, the protective effects of DCHD-DS on TLR4 and its downstream proteins were not enhanced.

**Fig. 12:**
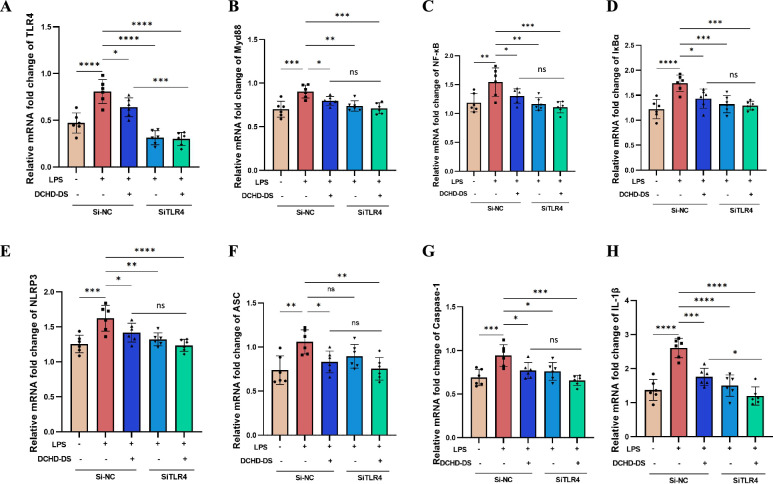
Effect of DCHD-DS on NF-κB/NLRP3/Caspase-1 signalling in LPS-induced ImKCs after siTLR4 transfection. **A–H** mRNA expression levels of *TLR4*, *Myd88*, *IκBα*, *NF-κB p65*, *NLRP3*, *ASC*, *Caspase-1*, and *IL-1β* in ImKCs (*n* = 6). Date are presented as the mean ± SD. *p < 0.05, **p < 0.01, ***p < 0.001, and ****p < 0.0001

To establish whether the anti-inflammatory effect of DCHD-DS was ultimately mediated through the suppression of the NLRP3 inflammasome, we used MCC950, a specific pharmacological inhibitor of NLRP3. Western blot analysis was performed to evaluate the expression of key proteins associated with the NLRP3/Caspase-1/IL-1β pathway. We found that the levels of NLRP3, ASC, Cleaved Caspased-1, and IL-1β were markedly elevated in the model group compared to those in the control group (*p* < 0.01), which could be reduced by MCC950 intervention (*p* < 0.05). Interestingly, DCHD-DS treatment exerted an inhibitory effect comparable to that of MCC950 (*p* < 0.05). To determine whether DCHD-DS and MCC950 act through the same pathway, we next examined their combined effect. Statistical analysis revealed no significant interaction between MCC950 and DCHD-DS treatment (*p* > 0.05), and no additional reduction in the levels of the NLRP3 inflammasome or its downstream proteins was observed following DCHD-DS treatment in the presence of NLRP3 inhibition (Fig. 11L and M). To further validate the involvement of the NF-κB/NLRP3/Caspase-1 signaling axis in mediating the effects of DCHD, we performed loss‑of‑function experiments using siRNA targeting TLR4. Following siTLR4 transfection, the mRNA expression levels of *TLR4*, *Myd88*, *NF‑κB‑p65*, *IκBα*, *NLRP3*, *Caspase-1*, *ASC* and *IL-1β* were quantified by qRT‑PCR (Fig. 12). Compared with the si-NC + LPS group, the mRNA expression level of *TLR4* was significantly decreased in siTLR4 + LPS group (*p* < 0.0001), the results revealed that knock-down TLR4 was effective. Then, compared with the si-NC + LPS group, DCHD-DS significantly decreased the mRNA expression levels of *TLR4*, *Myd88*, *NF‑κB‑p65*, *IκBα*, *NLRP3*, *Caspase-1*, *ASC* and *IL-1β* (*p* < 0.05). Moreover, Compared with the si-NC + LPS + DCHD-DS group, the mRNA expression levels of *TLR4*, *MyD88*, *NF-κB p65*, *IκBα*, *NLRP3*, *Caspase-1*, and *ASC* were not significantly decreased in the siTLR4 + LPS + DCHD-DS group (*p* > 0.05), whereas the mRNA level of *IL-1β* was significantly decreased. These findings indicate that DCHD-DS suppresses LPS-induced inflammatory injury in ImKCs may primarily through the NF-κB/NLRP3/Caspase-1 signaling pathway.

## Discussion

The management of SLI poses a major clinical challenge due to the limited availability of effective therapies. Dysbiosis of the gut microbiota can potentiate SLI progression by compromising intestinal barrier [47]. Therefore, probiotics and fecal microbiota transplantation are emerging as promising therapeutic avenues for SLI [48]. A growing body of evidence indicates that TCM can ameliorate SLI by modulating gut microbiota and enhancing intestinal barrier function, thereby highlighting its distinct therapeutic advantages [49, 50]. In this study, we employed an integrative approach combining 16S rRNA sequencing, network pharmacology, and liver transcriptomics to investigate the efficacy and mechanisms of action of DCHD in SLI. Our results demonstrated that DCHD effectively alleviated acute liver injury by reshaping the gut microbiota structure, restoring intestinal barrier integrity, and suppressing enteric translocation of LPS to the liver in CLP-induced septic mice. Consequently, these actions led to the inhibition of the hepatic TLR4/NF-κB/NLRP3/Caspase-1 signaling pathway, which was reflected by decreased serum ALT and AST levels and attenuated histopathological damage in liver tissues. Collectively, these findings not only validate the therapeutic potential of DCHD in sepsis-associated liver dysfunction by elucidating its multi-targeted mechanism but also provide experimental evidence supporting the use of TCM in treating liver diseases through modulation of gut homeostasis, thereby laying a robust scientific foundation for its therapeutic application.

DCHD is extensively used in clinical settings for the management of infectious diseases. Previous studies involving both cell and animal models have corroborated its anti-inflammatory potential, as demonstrated in the present study. In a rat model of sodium taurocholate-induced severe acute pancreatitis, the administration of DCHD markedly suppressed NF-κB expression, thereby attenuating inflammatory responses in the lung, ileum, and pancreas [51]. Furthermore, in mice with cerulein-induced chronic pancreatitis, DCHD treatment corresponded with inhibition of the MAPK signaling pathway, leading to the alleviation of pancreatic inflammatory cell infiltration and fibrosis [52]. DCHD is a promising therapeutic candidate for sepsis, owing to its potent anti-inflammatory and antioxidant properties. In the present study, a murine model of sepsis was established using CLP, which is currently the most extensively used experimental approach and is widely recognized to closely mimic the pathophysiology of human sepsis [53]. We observed that sepsis led to markedly elevated levels of pro-inflammatory cytokines, extensive inflammatory cell infiltration in hepatic tissue, and substantial hepatocyte apoptosis and oxidative stress injury, collectively resulting in severe hepatic dysfunction. Notably, these pathophysiological alterations were effectively reversed following the DCHD intervention. These findings underscore the therapeutic potential of traditional Chinese medicine, particularly DCHD, in the management of sepsis, although the underlying mechanisms warrant further investigation.

The interplay between the gut microbiota and the pathogenesis of sepsis is increasingly being recognized. Consequently, targeting the intestinal microbiome is a promising strategy for developing novel sepsis therapies [54, 55]. Notably, DCHD, a classic formulation grounded in the TCM theory of “Liver-Intestine Interaction” [21], has been shown to ameliorate non-alcoholic fatty liver disease by remodeling the gut microbiota in rats [17]. After oral administration, Chinese herbal compounds follow a dual-pathway distribution: certain active components are absorbed into the systemic circulation, whereas others remain within the gastrointestinal tract to directly modulate the gut microbial ecosystem [56]. To elucidate the mechanism of DCHD in SLI, we adopted a novel approach to investigate its role from a microbiological perspective. In the present study, CLP-induced septic mice exhibited substantial alterations in the gut microbiota architecture compared to the sham group, characterized by reduced richness and diversity, a decline in beneficial commensals (e.g., *Akkermansia*), and expansion of potential pathogens (e.g., *Klebsiella* and *Enterobacter*). Consistent with this dysbiosis, we observed compromised TJ barrier integrity, as evidenced by the downregulated expression of ZO-1, E-cadherin, and occludin, corroborating previous reports [41, 57]. As integral components of the intestinal barrier, TJ proteins inhibit the translocation of bacteria and toxins via paracellular and trans-epithelial routes, thereby preserving the gut permeability and epithelial barrier integrity [58]. Intriguingly, intervention with DCHD protected intestinal barrier function in septic mice by promoting microbial diversity, increasing the proportion of beneficial bacteria, and enhancing the expression of tight-junction proteins. *Akkermansia*, a Gram-negative anaerobic bacterium regarded as the next-generation probiotic, is of particular interest. It demonstrates considerable potential for correcting gut dysbiosis and intestinal barrier dysfunction, showing promise for sepsis management [59–61]. Given that members of the order *Lactobacillales* were identified as the signature microbiota in DCHD-treated mice, this finding is significant from prior research demonstrating that *Lactobacillus* can reverse sepsis-induced dysbiosis and ameliorate multiorgan injury by modulating hepatic inflammatory immune responses [49, 62]. Therefore, *Akkermansia* and *Lactobacillus* have emerged as promising probiotic candidates for SLI treatment. Driven by a growing appreciation for complex host-microbiota dialogue, scientific inquiry is moving beyond correlation to dissect the specific functions of individual bacterial species.

Probiotics mediate their beneficial effects via multiple mechanisms such as direct interaction with host cells, production of bioactive metabolites, remodeling of the host microbial ecosystem, and immunomodulation [63]. Our findings demonstrated that D-lactate and DAO, serum markers of intestinal permeability [60], were significantly elevated in CLP-induced sepsis mice, suggesting severe intestinal epithelial damage, loss of mucosal integrity, and increased intestinal permeability. Upon loss of gut barrier integrity, bacterial metabolites, such as LPS, can translocate into the bloodstream and subsequently reach distant organs [63, 64]. Consequently, identifying and characterizing these active metabolites is crucial for understanding how DCHD modulates gut microbiota to influence SLI. In our study, serum LPS levels were significantly elevated in septic mice and positively correlated with the abundance of *Klebsiella*, *Enterobacter*, and *Proteus*, whereas DCHD intervention reversed these changes. LPS, a component of the outer membrane of Gram-negative bacteria, is released upon bacterial death or division. Following its release, LPS can translocate across the compromised intestinal barrier via pathways such as chylomicrons to enter the portal circulation and then be transported to the liver and cleared by KCs [65, 66]. Based on these findings, we postulate that CLP-induced sepsis leads to gut microbiota dysbiosis, characterized by the expansion of gram-negative bacteria such as *Klebsiella*, *Enterobacter*, and *Proteus*. The subsequent increase in LPS translocation to the liver via the enterohepatic circulation triggers a cascade of local inflammatory and immune responses. Notably, we observed significant upregulation of KC-specific markers (CD11B and F4/80) in septic mice. This finding partially validates our hypothesis; however, further verification using fecal microbiota transplantation (FMT) is required.

Chinese herbal formulae typically exert therapeutic effects through multi-component and multi-target mechanisms. In the present study, we performed a chemical characterization of DCHD using mass spectrometry (MS) for quality control. Subsequent network pharmacology analysis identified Genistein, Wogonin, and Kaempferol as the potential core active components. Previous studies have demonstrated that genistein alleviates alcohol-induced liver injury through antioxidant, anti-inflammatory, and anti-apoptotic mechanisms [67]. Additionally, Wogonin ameliorated SLI by activating Nrf2-mediated transcription, thereby simultaneously suppressing oxidative stress and inflammatory responses [68]. Similarly, kaempferol has been shown to have protective efficacy against sepsis [69]. These findings collectively provide valuable insights and a foundational framework for the current investigation. In addition, integrated network pharmacology and molecular docking analyses identified ALB, CASP3, AKT1, MMP9, PPARG, HSP90AB1, SRC, EGFR, and IGF1 as core targets of DCHD in SLE treatment. Notably, the core bioactive components, genistein, wogonin, and kaempferol demonstrated robust binding affinities for ALB, MMP9, and HSP90AB1. These specific targets have been previously established as critical players in inflammation, oxidative stress, and the regulation of cell proliferation and apoptosis [70–73]. Collectively, these findings underscore that DCHD likely exerts its therapeutic effects by simultaneously targeting multiple pathways to inhibit inflammation, oxidative stress, and apoptosis. Similarly, hepatic transcriptomics revealed that some pro-inflammatory cytokines (e.g., IL-6, IL-1β, CCL2, and CXCL5) were identified as core genes. This finding provides compelling experimental validation for prior inferences drawn from our network pharmacological analysis.

Integrated analysis of network pharmacology and hepatic transcriptomics identified the canonical NF-κB signaling pathway as one of the key pathways through which DCHD treats SLI. This finding corroborates our earlier hypothesis regarding hepatic translocation of gut-derived LPS. Upon translocation to the liver, LPS functions as a PAMP recognized by TLR4 in hepatic immune cells, including KCs [65]. This recognition triggers the activation of the downstream NF-κB signaling pathway. NF-κB activation not only promotes the transcription of classical inflammatory cytokines such as TNF-α and IL-6, but also initiates the expression of both the NLRP3 inflammasome and its substrate precursor, caspase-1. This process primes the inflammasome for subsequent “assembly” and “activation” [74, 75]. According to previous studies, the activation of the NF-κB signaling pathway promotes the upregulation of NLRP3 expression, which in turn facilitates the recruitment of the adaptor protein ASC and pro-caspase-1. This leads to the assembly of a high-molecular-weight complex, in which caspase-1 is activated. Activated caspase-1 subsequently cleaves both GSDMD and the precursor cytokine pro-IL-1β to generate mature IL-1β. Cleaved GSDMD forms pores in the plasma membrane, enabling the release of cytoplasmic contents and ultimately triggering inflammatory responses [76–78]. During sepsis, PAMPs such as LPS activate TLR4, triggering the MyD88‑dependent NF‑κB pathway. This leads to phosphorylation and degradation of IκBα, allowing nuclear translocation of NF‑κB p65 and subsequent transcriptional upregulation of pro‑IL‑1β and NLRP3, thereby providing the priming signal for inflammasome activation, thereby driving robust inflammatory responses and hepatocyte pyroptosis, ultimately contributing to liver dysfunction. Thus, by inhibiting TLR4, suppressing NF-κB activation, or blocking NLRP3 inflammasome assembly—represents a promising strategy to attenuate hepatic inflammation and improve outcomes in SLI [79, 80]. In a previous study, Qingyi decoction, along with its primary active constituent wogonoside, was demonstrated to effectively ameliorate acute pancreatitis by targeting the NF-κB/NLRP3/caspase-1 signaling pathway [81]. Moreover, Genistein, Wogonin, and Kaempferol, key active compounds in DCHD, have been reported to alleviate liver injury through suppression of inflammation via NF-κB related pathways [82–84]. Comprehensive in vivo and in vitro experiments were conducted to validate these findings. In vivo studies demonstrated that DCHD effectively suppressed the activation of NF-κB and NLRP3 inflammasomes, significantly reducing the expression of pro-inflammatory cytokines. For the in vitro investigation, we established a hepatic inflammatory injury model by stimulating ImKCs with LPS, followed by treatment with DCHD-DS, the TLR4 inhibitor TAK-242, or the NLRP3 inhibitor MCC950. Consistent with the in vivo results, DCHD-DS treatment markedly inhibited the upregulation of p-NF-κB p65, p-IκBα, NLRP3, ASC, and Cleaved Caspase-1 in ImKCs, while concurrently reducing IL-1β expression.

Despite these advances, this study had several limitations. First, a murine model of sepsis was established using the widely recognized CLP method in the current study. In comparison, the LPS injection-induced sepsis model offers high reproducibility and demonstrates notable efficacy for inducing inflammatory responses. Therefore, the concurrent utilization of both models is recommended to substantially strengthen the validity of the research findings. Secondly, the composition of DCHD is complex, and the key active components responsible for inhibiting the NF-κB pathway and suppressing NLRP3 inflammasome activation to ameliorate SLI remain unclear. The inclusion of an appropriate positive control would serve as an important benchmark, enabling a more robust comparison of the efficacy of DCHD on CLP-induced SLI in mice, thereby enhancing the robustness and credibility of the study findings. Third, given the substantial mortality observed within 24 h in the CLP-induced sepsis model, we adopted a short treatment period with uniform samples collection at 24 h post-surgery. This approach may introduce survivor bias. Future studies should consider initiating drug administration 5–7 days before modeling and collecting samples at multiple early time points (e.g., 6 and 12 h) to better assess the therapeutic efficacy of DCHD against SLI. Moreover, this study lacks functional validation experiments such as FMT or gene knockout models, which are essential for establishing definitive causal relationships. Future studies should prioritize addressing these issues to substantiate the proposed conclusions further.

## Conclusion

**Fig. 13:**
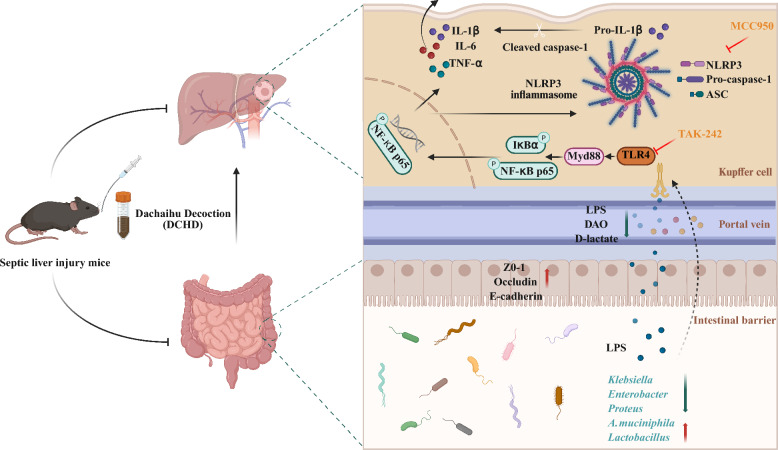
DCHD alleviates SLI by modulating the intestinal barrier dysfunction and suppressing the NF-κB/NLRP3/Caspase-1 signaling pathway

In conclusion, we employed an integrated approach combining network pharmacology, transcriptomics, and 16S rRNA sequencing to investigate the mechanisms by which DCHD ameliorates SLI. Our findings indicate that DCHD potentially attenuates hepatic inflammatory damage by remodeling the gut microbiota structure and diversity in mice with CLP-induced sepsis, thereby enhancing intestinal barrier integrity. Consequently, this intervention impedes the translocation of gut-derived LPS to the liver and suppresses the activation of the hepatic NF-κB/NLRP3/caspase-1 signaling pathway (Fig. 13). These insights reveal the underlying mechanisms through which DCHD may alleviate sepsis-associated liver injury, warranting further validation via FMT and exogenous LPS supplementation. Collectively, this study provides compelling preclinical evidence supporting the use of DCHD as a promising herbal formulation for sepsis treatment, thereby laying a solid foundation for subsequent investigations.

## Supplementary Information

Additional file1 (XLSX 21 KB)

Additional file2 (DOCX 1175 KB)

Additional file3 (XLSX 221 KB)

Additional file4 (DOCX 13 KB)

## Data Availability

The data that support the findings of this study are available from the corresponding author upon reasonable request.

## Abbreviations

ALT: Alanine aminotransferase
ASC: Apoptosis- associated speck-like protein containing a CARD
AST: Aspartate aminotransferase
ANOVA: One-way analysis of variance
ASVs: Amplicon sequence variants
BP: Biological process
Caspase-1: Cysteine-aspartic acid protease 1
CAT: Catalase
CC: Cellular component
CLP: Cecal ligation and puncture
DAB: Diaminobenzidine
DAO: Diamine oxidase
DCHD: Dachaihu decoction
DCHD-DS: DCHD drug containing serum
DEGs: Differentially expressed genes
EPC: Edge percolated component
GEO: Gene expression omnibus
GO: Gene Ontology
GSH-Px: Glutathione peroxidas
H&E: Hematoxylin and eosin
ImKCs: Immortalized mouse Kupffer cells
IL-6: Interleukin-6
KCs: Kupffer cells
KEGG: Kyoto encyclopedia of genes and genomes
LEfSe: Linear discriminant analysis effect size
LPS: Lipopolysaccharide
LSD: Least significant difference
MCC: Maximal clique centrality
MDA: Malondialdehyde
MF: Molecular Function
MNC: Maximum neighborhood component
MODS: Multiple organ dysfunction syndrome
NF-κB: Nuclear factor-κB
NLRP3: NOD-like receptor protein 3
MSS: Murine sepsis score
PAMP: Pathogen-associated molecular pattern
PCA: Principal component analysis
PFA: Paraformaldehyde
PLS-DA: Partial least squares discriminant analysis
PPI: Protein-protein interaction
PVDF: Polyvinylidene fluoride
qRT-PCR: Quantitative real-time PCR
SD: Sprague–Dawley
SLI: Septic liver injury
SOD: Superoxide dismutase
SD: Standard deviation
SPF: Specific pathogen-free
TCM: Traditional Chinese medicine
TEM: Transmission electron microscopy
TJs: Tight junctions
TLR4: Toll-like receptor-4
TNF-α: Tumor necrosis factor-α
TUNEL: Terminal deoxynucleotidyl transferase dUTP nick end labeling
UPLC-Q-TOF–MS: Ultra-high performance liquid chromatography with quadrupole time-of-flight mass spectrometry
ZO-1: Zonula occludens-1

## References

[1] Shankar-Hari M, Phillips GS, Levy ML, Seymour CW, Liu VX, Deutschman CS, et al. Developing a new definition and assessing new clinical criteria for septic shock: for the Third International Consensus Definitions for Sepsis and Septic Shock (Sepsis-3). JAMA. 2016;315:775–87. 26903336 10.1001/jama.2016.0289 PMC4910392

[2] Rudd KE, Johnson SC, Agesa KM, Shackelford KA, Tsoi D, Kievlan DR, et al. Global, regional, and national sepsis incidence and mortality, 1990-2017: analysis for the Global Burden of Disease Study. Lancet. 2020;395:200–11. 31954465 10.1016/S0140-6736(19)32989-7 PMC6970225

[3] Elias G, Schonfeld M, Saleh S, Parrish M, Barmanova M, Weinman SA, et al. Sepsis-induced endothelial dysfunction drives acute-on-chronic liver failure through Angiopoietin-2-HGF-C/EBPbeta pathway. Hepatology. 2023;78:803–19. 36943063 10.1097/HEP.0000000000000354 PMC10440279

[4] Kobashi H, Toshimori J, Yamamoto K. Sepsis-associated liver injury: incidence, classification and the clinical significance. Hepatol Res. 2013;43:255–66. 22971102 10.1111/j.1872-034X.2012.01069.x

[5] Yan J, Li S, Li S. The role of the liver in sepsis. Int Rev Immunol. 2014;33:498–510. 24611785 10.3109/08830185.2014.889129 PMC4160418

[6] Lelubre C, Vincent JL. Mechanisms and treatment of organ failure in sepsis. Nat Rev Nephrol. 2018;14:417–27. 29691495 10.1038/s41581-018-0005-7

[7] Chen JW, Liu CY, Li S, Wu SW, Cai C, Lu MQ. Sepsis-associated liver injury: mechanisms and potential therapeutic targets. World J Gastroenterol. 2024;30:4518–22. 39563749 10.3748/wjg.v30.i42.4518 PMC11572628

[8] Luo D, Luo G, Xu H, Li K, Li Z, Zhang C. Inorganic dietary nanoparticles in intestinal barrier function of inflammatory bowel disease: allies or adversaries? Front Immunol. 2025;16:1563504. 40270957 10.3389/fimmu.2025.1563504 PMC12014688

[9] Klingensmith NJ, Coopersmith CM. The gut as the motor of multiple organ dysfunction in critical illness. Crit Care Clin. 2016;32:203–12. 27016162 10.1016/j.ccc.2015.11.004 PMC4808565

[10] Miele L, Valenza V, La Torre G, Montalto M, Cammarota G, Ricci R, et al. Increased intestinal permeability and tight junction alterations in nonalcoholic fatty liver disease. Hepatology. 2009;49:1877–87. 19291785 10.1002/hep.22848

[11] Allaire JM, Crowley SM, Law HT, Chang SY, Ko HJ, Vallance BA. The intestinal epithelium: central coordinator of mucosal immunity. Trends Immunol. 2018;39:677–96. 29716793 10.1016/j.it.2018.04.002

[12] Chelakkot C, Ghim J, Ryu SH. Mechanisms regulating intestinal barrier integrity and its pathological implications. Exp Mol Med. 2018;50:1–9. 30115904 10.1038/s12276-018-0126-x PMC6095905

[13] Zhang X, Liu H, Hashimoto K, Yuan S, Zhang J. The gut-liver axis in sepsis: interaction mechanisms and therapeutic potential. Crit Care. 2022;26:213. 35831877 10.1186/s13054-022-04090-1 PMC9277879

[14] Cani PD, Amar J, Iglesias MA, Poggi M, Knauf C, Bastelica D, et al. Metabolic endotoxemia initiates obesity and insulin resistance. Diabetes. 2007;56:1761–72. 17456850 10.2337/db06-1491

[15] Song Y, Lin W, Zhu W. Traditional Chinese medicine for treatment of sepsis and related multi-organ injury. Front Pharmacol. 2023;14:1003658. 36744251 10.3389/fphar.2023.1003658 PMC9892725

[16] Xie L, Zhou C, Wu Y, Fu X, Zhang G, Han X, et al. Wenqingyin suppresses ferroptosis in the pathogenesis of sepsis-induced liver injury by activating the Nrf2-mediated signaling pathway. Phytomedicine. 2023;114:154748. 36933519 10.1016/j.phymed.2023.154748

[17] Cui H, Li Y, Wang Y, Jin L, Yang L, Wang L, et al. Da-Chai-Hu decoction ameliorates high fat diet-induced nonalcoholic fatty liver disease through remodeling the gut microbiota and modulating the serum metabolism. Front Pharmacol. 2020;11:584090. 33328987 10.3389/fphar.2020.584090 PMC7732620

[18] Zhou Y, Zhou Y, Li Y, Sun W, Wang Z, Chen L, et al. Targeted bile acid profiles reveal the liver injury amelioration of Da-Chai-Hu decoction against ANIT- and BDL-induced cholestasis. Front Pharmacol. 2022;13:959074. 36059946 10.3389/fphar.2022.959074 PMC9437253

[19] Duan LF, Xu XF, Zhu LJ, Liu F, Zhang XQ, Wu N, et al. Dachaihu decoction ameliorates pancreatic fibrosis by inhibiting macrophage infiltration in chronic pancreatitis. World J Gastroentero. 2017;23:7242–52. 10.3748/wjg.v23.i40.7242 PMC5677205 29142471

[20] Xu XF, Jiang TT, Liu F, Zhang XQ, Shi YL, Chen Y, et al. Effect of DaChaiHu Decoction on pancreatic fibrosis induced by DBTC combined with alcohol and the mechanism of TGF-beta/Smad signaling pathway. Zhongguo Ying Yong Sheng Li Xue Za Zhi. 2016;32:444–8. 29931849 10.13459/j.cnki.cjap.2016.05.015

[21] Yang Z, Kao X, Zhu T, Lei J, Huang N, Chen J, et al. Clinical efficacy and metabolomics profiling of Dachaihu decoction for patients with septic liver injury: a randomized controlled trial. Front Pharmacol. 2025;16:1671732. 41378216 10.3389/fphar.2025.1671732 PMC12685928

[22] Yang Z, Kao X, Zhang L, Huang N, Chen J, He M. Exploring the Anti-PANoptosis mechanism of Dachaihu decoction against sepsis-induced acute lung injury: network pharmacology, bioinformatics, and experimental validation. Drug Des Devel Ther. 2025;19:349–68. 39839500 10.2147/DDDT.S495225 PMC11750123

[23] Bochu W, Liancai Z, Qi C. Primary study on the application of serum pharmacology in Chinese traditional medicine. Colloids Surf B Biointerfaces. 2005;43:194–7. 15964749 10.1016/j.colsurfb.2005.04.013

[24] Wang Y, Zhang J, Zhang B, Lu M, Ma J, Liu Z, et al. Modified Gegen Qinlian decoction ameliorated ulcerative colitis by attenuating inflammation and oxidative stress and enhancing intestinal barrier function in vivo and in vitro. J Ethnopharmacol. 2023;313:116538. 37086872 10.1016/j.jep.2023.116538

[25] Ru J, Li P, Wang J, Zhou W, Li B, Huang C, et al. TCMSP: a database of systems pharmacology for drug discovery from herbal medicines. J Cheminform. 2014;6:13. 24735618 10.1186/1758-2946-6-13 PMC4001360

[26] Daina A, Michielin O, Zoete V. SwissTargetPrediction: updated data and new features for efficient prediction of protein targets of small molecules. Nucleic Acids Res. 2019;47:W357–64. 31106366 10.1093/nar/gkz382 PMC6602486

[27] Barrett T, Wilhite SE, Ledoux P, Evangelista C, Kim IF, Tomashevsky M, et al. NCBI GEO: archive for functional genomics data sets--update. Nucleic Acids Res. 2013;41:D991–5. 23193258 10.1093/nar/gks1193 PMC3531084

[28] Xiao C, Wang Y, Liu J, Li X, Wang P, Zhou J, et al. Mechanism of Fangji Huangqi decoction against acute kidney injury based on network pharmacology and experimental validation. Phytomedicine. 2025;136:156345. 39742571 10.1016/j.phymed.2024.156345

[29] Rittirsch D, Huber-Lang MS, Flierl MA, Ward PA. Immunodesign of experimental sepsis by cecal ligation and puncture. Nat Protoc. 2009;4:31–6. 19131954 10.1038/nprot.2008.214 PMC2754226

[30] Shrum B, Anantha RV, Xu SX, Donnelly M, Haeryfar SM, McCormick JK, et al. A robust scoring system to evaluate sepsis severity in an animal model. BMC Res Notes. 2014;7:233. 24725742 10.1186/1756-0500-7-233 PMC4022086

[31] Jin GL, Liu HP, Huang YX, Zeng QQ, Chen JX, Lan XB, et al. Koumine regulates macrophage M1/M2 polarization via TSPO, alleviating sepsis-associated liver injury in mice. Phytomedicine. 2022;107:154484. 36215787 10.1016/j.phymed.2022.154484

[32] Han S, Zhang H, Qian J, Yao S, Sun Y, Zhao X, et al. Qiangxin bushen decoction attenuates cardiorenal syndrome type II via AMPK/FOXO1-mediated ferroptosis pathway: a multi-omics and experimental study. Phytomedicine. 2025;147:157247. 40961594 10.1016/j.phymed.2025.157247

[33] Huang N, Wei Y, Wang M, Liu M, Kao X, Yang Z, et al. Dachaihu decoction alleviates septic intestinal epithelial barrier disruption via PI3K/AKT pathway based on transcriptomics and network pharmacology. J Ethnopharmacol. 2025;337:118937. 39419306 10.1016/j.jep.2024.118937

[34] Gong S, Yan Z, Liu Z, Niu M, Fang H, Li N, et al. Intestinal microbiota mediates the susceptibility to polymicrobial sepsis-induced liver injury by Granisetron generation in mice. Hepatology. 2019;69:1751–67. 30506577 10.1002/hep.30361

[35] Zhang L, Gui S, Liang Z, Liu A, Chen Z, Tang Y, et al. *Musca domestica* cecropin (Mdc) alleviates *Salmonella typhimurium*-induced colonic mucosal barrier impairment: associating with inflammatory and oxidative stress response, tight junction as well as intestinal flora. Front Microbiol. 2019;10:522. 30930887 10.3389/fmicb.2019.00522 PMC6428779

[36] Emu Q, Guan H, Zhu J, Zhang L, Fan J, Ji Y, et al. Grazing and supplementation of dietary yeast probiotics shape the gut microbiota and improve the immunity of black fattening goats (*Capra hircus*). Front Microbiol. 2021;12:666837. 34489878 10.3389/fmicb.2021.666837 PMC8416523

[37] Chen G, Wang W, Guan B, Zhang G, Zhang Z, Lin L, et al. Cycloastragenol reduces inflammation in CLP-induced septic mice by suppressing TLR4 signaling pathways. Phytomedicine. 2025;142:156645. 40319834 10.1016/j.phymed.2025.156645

[38] Liu L, Zhou L, Wang L, Mao Z, Zheng P, Zhang F, et al. MUC1 attenuates neutrophilic airway inflammation in asthma by reducing NLRP3 inflammasome-mediated pyroptosis through the inhibition of the TLR4/MyD88/NF-kappaB pathway. Respir Res. 2023;24:255. 37880668 10.1186/s12931-023-02550-y PMC10601133

[39] Li X, Yao M, Li L, Ma H, Sun Y, Lu X, et al. Aloe-emodin alleviates cerebral ischemia-reperfusion injury by regulating microglial polarization and pyroptosis through inhibition of NLRP3 inflammasome activation. Phytomedicine. 2024;129:155578. 38621328 10.1016/j.phymed.2024.155578

[40] Fang R, Zhou R, Ju D, Li M, Wang H, Pan L, et al. Zhen-wu-tang protects against myocardial fibrosis by inhibiting M1 macrophage polarization via the TLR4/NF-κB pathway. Phytomedicine. 2024;130:155719. 38763013 10.1016/j.phymed.2024.155719

[41] Huang W, Chen H, He Q, Xie W, Peng Z, Ma Q, et al. Nobiletin protects against ferroptosis to alleviate sepsis-associated acute liver injury by modulating the gut microbiota. Food Funct. 2023;14:7692–704. 37545398 10.1039/d3fo01684f

[42] Wang H, Liu D. Baicalin inhibits high-mobility group box 1 release and improves survival in experimental sepsis. Shock. 2014;41:324–30. 24430548 10.1097/SHK.0000000000000122

[43] Wu B, Song H, Fan M, You F, Zhang L, Luo J, et al. Luteolin attenuates sepsis‑induced myocardial injury by enhancing autophagy in mice,. Int J Mol Med. 2020;45:1477–87. 32323750 10.3892/ijmm.2020.4536 PMC7138288

[44] Strnad P, Tacke F, Koch A, Trautwein C. Liver—guardian, modifier and target of sepsis,. Nat Rev Gastroenterol Hepatol. 2017;14:55–66. 27924081 10.1038/nrgastro.2016.168

[45] Xie L, Zhang G, Wu Y, Hua Y, Ding W, Han X, et al. Protective effects of Wenqingyin on sepsis-induced acute lung injury through regulation of the receptor for advanced glycation end products pathway,. Phytomedicine. 2024;129:155654. 38723525 10.1016/j.phymed.2024.155654

[46] Luo R, Yao Y, Chen Z, Sun X. An examination of the LPS-TLR4 immune response through the analysis of molecular structures and protein-protein interactions,. Cell Commun Signal. 2025;23:142. 40102851 10.1186/s12964-025-02149-4 PMC11921546

[47] Shahid A, Chambers S, Scott-Thomas A, Bhatia M. Gut microbiota and liver dysfunction in sepsis: the role of inflammatory mediators and therapeutic approaches. Int J Mol Sci. 2024;25:13415. 39769181 10.3390/ijms252413415 PMC11678143

[48] Liang H, Song H, Zhang X, Song G, Wang Y, Ding X, et al. Metformin attenuated sepsis-related liver injury by modulating gut microbiota,. Emerg Microbes Infect. 2022;11:815–28. 35191819 10.1080/22221751.2022.2045876 PMC8928825

[49] Fan X, Mai C, Zuo L, Huang J, Xie C, Jiang Z, et al. Herbal formula BaWeiBaiDuSan alleviates polymicrobial sepsis-induced liver injury via increasing the gut microbiota *Lactobacillus johnsonii* and regulating macrophage anti-inflammatory activity in mice,. Acta Pharm Sin B. 2023;13:1164–79. 36970196 10.1016/j.apsb.2022.10.016 PMC10031256

[50] Wang Z, Lin X, Wu J, Su C, Luo Y, Yu G. Radix Pseudostellaria polysaccharides alleviate sepsis-induced liver injury by modulating the gut microbiota via the TLR4/NF-kappaB pathway,. Front Pharmacol. 2025;16:1658147. 41069598 10.3389/fphar.2025.1658147 PMC12504199

[51] Zhao G, Zhuo YZ, Cui LH, Li CX, Chen SY, Li D, et al. Modified Da-chai-hu decoction regulates the expression of occludin and NF-kappaB to alleviate organ injury in severe acute pancreatitis rats. Chin J Nat Med. 2019;17:355–62. 31171270 10.1016/S1875-5364(19)30041-X

[52] Li X, Yan Z, Cao X, Chen X, Guan Z, Tang S, et al. Dachaihu decoction alleviates chronic pancreatitis by regulating MAPK signaling pathway: insights from network pharmacology and experimental validation. J Ethnopharmacol. 2025;337:118833. 39306212 10.1016/j.jep.2024.118833

[53] Zhu B, Sun C, Luo D, Liang Y, Jiang A, Jiang Z, et al. Coptisine improves liver inflammation in sepsis by regulating STAT1/IRF1/GPX4 signaling-mediated Kupffer cells ferroptosis. Phytother Res. 2025;39:4308–26. 40791017 10.1002/ptr.70063

[54] Miller WD, Keskey R, Alverdy JC. Sepsis and the microbiome: a vicious cycle. J Infect Dis. 2021;223:S264–9. 33330900 10.1093/infdis/jiaa682 PMC8206800

[55] Gai X, Wang H, Li Y, Zhao H, He C, Wang Z, et al. Fecal microbiota transplantation protects the intestinal mucosal barrier by reconstructing the gut microbiota in a murine model of sepsis. Front Cell Infect Microbiol. 2021;11:736204. 34631604 10.3389/fcimb.2021.736204 PMC8493958

[56] Liu Y, Zhao P, Cai Z, He P, Wang J, He H, et al. Buqi-Huoxue-Tongnao decoction drives gut microbiota-derived indole lactic acid to attenuate ischemic stroke via the gut-brain axis. Chin Med Lond. 2024;19:126. 10.1186/s13020-024-00991-1 PMC11403783 39278929

[57] Niu M, Chen P. Crosstalk between gut microbiota and sepsis. Burns Trauma. 2021;9:tkab36. 10.1093/burnst/tkab036 PMC8547143 34712743

[58] Pabst O, Hornef MW, Schaap FG, Cerovic V, Clavel T, Bruns T. Gut-liver axis: barriers and functional circuits. Nat Rev Gastroenterol Hepatol. 2023;20:447–61. 37085614 10.1038/s41575-023-00771-6

[59] Liu J, Liu Y, Huang C, He C, Yang T, Ren R, et al. Quercetin-driven *Akkermansia muciniphila* alleviates obesity by modulating bile acid metabolism via an ILA/m(6)A/CYP8B1 signaling. Adv Sci. 2025;12:e2412865. 10.1002/advs.202412865 PMC11948036 39888270

[60] Wang J, Peng Y, Liu Y, Lian Z, Cai Z, Chen Y, et al. Indole lactic acid derived from *Akkermansia muciniphila* activates the aryl hydrocarbon receptor to inhibit ferroptosis in ischemic stroke. Free Radic Biol Med. 2025;234:113–30. 40246252 10.1016/j.freeradbiomed.2025.04.020

[61] Xie S, Li J, Lyu F, Xiong Q, Gu P, Chen Y, et al. Novel tripeptide RKH derived from *Akkermansia muciniphila* protects against lethal sepsis. Gut. 2023;73:78–91. 37553229 10.1136/gutjnl-2023-329996

[62] Chen L, Li H, Li J, Chen Y, Yang Y. *Lactobacillus rhamnosus* GG treatment improves intestinal permeability and modulates microbiota dysbiosis in an experimental model of sepsis. Int J Mol Med. 2019;43:1139–48. 30628657 10.3892/ijmm.2019.4050 PMC6365041

[63] Maftei NM, Raileanu CR, Balta AA, Ambrose L, Boev M, Marin DB, et al. The potential impact of probiotics on human health: an update on their health-promoting properties. Microorganisms. 2024;12:234. 38399637 10.3390/microorganisms12020234 PMC10891645

[64] Deng W, Yi P, Xiong Y, Ying J, Lin Y, Dong Y, et al. Gut metabolites acting on the gut-brain axis: regulating the functional state of microglia. Aging Dis. 2024;15:480–502. 37548933 10.14336/AD.2023.0727 PMC10917527

[65] Li B, Jiang XF, Dong YJ, Zhang YP, He XL, Zhou CL, et al. The effects of *Atractylodes macrocephala* extract BZEP self-microemulsion based on gut-liver axis HDL/LPS signaling pathway to ameliorate metabolic dysfunction-associated fatty liver disease in rats. Biomed Pharmacother. 2024;175:116519. 38663104 10.1016/j.biopha.2024.116519

[66] Gu T, Kong M, Duan M, Chen L, Tian Y, Xu W, et al. Cu exposure induces liver inflammation via regulating gut microbiota/LPS/liver TLR4 signaling axis. Ecotox Environ Saf. 2024;278:116430. 10.1016/j.ecoenv.2024.116430 38718729

[67] Zhao L, Wang Y, Liu J, Wang K, Guo X, Ji B, et al. Protective effects of Genistein and Puerarin against chronic alcohol-induced liver injury in mice via antioxidant, anti-inflammatory, and anti-apoptotic mechanisms. J Agric Food Chem. 2016;64:7291–7. 27609057 10.1021/acs.jafc.6b02907

[68] Dai JM, Guo WN, Tan YZ, Niu KW, Zhang JJ, Liu CL, et al. Wogonin alleviates liver injury in sepsis through Nrf2-mediated NF-kappaB signalling suppression. J Cell Mol Med. 2021;25:5782–98. 33982381 10.1111/jcmm.16604 PMC8184690

[69] Zhu X, Wang X, Ying T, Li X, Tang Y, Wang Y, et al. Kaempferol alleviates the inflammatory response and stabilizes the pulmonary vascular endothelial barrier in LPS-induced sepsis through regulating the SphK1/S1P signaling pathway. Chem-Biol Interact. 2022;368:110221. 36243145 10.1016/j.cbi.2022.110221

[70] Chen Q, Zhan H, Chen J, Mo J, Huang S. Predictive value of lactate/albumin ratio for death and multiple organ dysfunction syndrome in patients with sepsis. J Med Biochem. 2024;43:617–25. 39139160 10.5937/jomb0-46947 PMC11318848

[71] Huang Y, Li G, Li D, Liu C, Chen M, Cai L, et al. Ethyl caffeate alleviates inflammatory response and promotes recovery in septic-acute lung injury via the TNF-alpha/NF-kappaB/MMP9 axis. Phytomedicine. 2025;141:156700. 40220405 10.1016/j.phymed.2025.156700

[72] Wu W, Wang J, Wang G, Wang F, Yang Y, Liu Z, et al. Monotropein inhibits MMP9-mediated cardiac oxidative stress, inflammation, matrix degradation and apoptosis in a mouse and cell line models of septic cardiac injury. Mol Biol Rep. 2025;52:329. 40111530 10.1007/s11033-025-10421-6

[73] Liu J, Yu H, Yu S, Liu M, Chen X, Wang Y, et al. GLCCI1 alleviates GRP78-initiated endoplasmic reticulum stress-induced apoptosis of retinal ganglion cells in diabetic retinopathy by upregulating and interacting with HSP90AB1. Sci Rep. 2024;14:26665. 39496608 10.1038/s41598-024-75874-4 PMC11535184

[74] Peng L, Wen L, Shi QF, Gao F, Huang B, Meng J, et al. Scutellarin ameliorates pulmonary fibrosis through inhibiting NF-kappaB/NLRP3-mediated epithelial-mesenchymal transition and inflammation. Cell Death Dis. 2020;11:978. 33188176 10.1038/s41419-020-03178-2 PMC7666141

[75] Wang Y, Zhang W, Yang Y, Qin J, Wang R, Wang S, et al. Osteopontin deficiency promotes cartilaginous endplate degeneration by enhancing the NF-kappaB signaling to recruit macrophages and activate the NLRP3 inflammasome. Bone Res. 2024;12:53. 39242551 10.1038/s41413-024-00355-3 PMC11379908

[76] Afonina IS, Zhong Z, Karin M, Beyaert R. Limiting inflammation-the negative regulation of NF-kappaB and the NLRP3 inflammasome. Nat Immunol. 2017;18:861–9. 28722711 10.1038/ni.3772

[77] Reid S, Scholey JW. Recent approaches to targeting canonical NFkappaB signaling in the early inflammatory response to renal IRI. J Am Soc Nephrol. 2021;32:2117–24. 34108233 10.1681/ASN.2021010069 PMC8729839

[78] Chawla M, Roy P, Basak S. Role of the NF-kappaB system in context-specific tuning of the inflammatory gene response. Curr Opin Immunol. 2021;68:21–7. 32898750 10.1016/j.coi.2020.08.005

[79] Zhong Z, Wang H, Liu X, Huang Q, Xie M, Xu H. Inhibition of AKR1C3 attenuates sepsis-induced acute liver injury by blocking the TRAF6/NF-κB pathway and NLRP3 inflammasome activation. Int Immunopharmacol. 2026;168:115859. 41270642 10.1016/j.intimp.2025.115859

[80] Liu A, Xiao L, He Q, Xu Z, Zhu Y, Zhang Z, et al. EA mitigates sepsis-induced liver injury by inhibiting the proinflammatory pathways IκBα/NF-κB and NLRP3-mediated pyroptosis. Microb Pathog. 2026;212:108309. 41579981 10.1016/j.micpath.2026.108309

[81] An Y, Tu Z, Wang A, Gou W, Yu H, Wang X, et al. Qingyi decoction and its active ingredients ameliorate acute pancreatitis by regulating acinar cells and macrophages via NF-kappaB/NLRP3/Caspase-1 pathways. Phytomedicine. 2025;139:156424. 40020626 10.1016/j.phymed.2025.156424

[82] Li S, Xie J, Li X, Li Q, Tang X, Yu D, et al. Genistein protects benzotriazole ultraviolet stabilizer UV-234-induced hepatotoxicity by modulating ROS/Nrf2 and NF-κB signaling in yellow catfish (*Pelteobagrus fulvidraco*). Comp Biochem Physiol C Toxicol Pharmacol. 2023;271:109675. 37269917 10.1016/j.cbpc.2023.109675

[83] Dai J, Guo W, Tan Y, Niu K, Zhang J, Liu C, et al. Wogonin alleviates liver injury in sepsis through Nrf2-mediated NF-κB signalling suppression. J Cell Mol Med. 2021;25:5782–98. 33982381 10.1111/jcmm.16604 PMC8184690

[84] Lee C, Yoon S, Moon J. Kaempferol suppresses Carbon Tetrachloride-induced liver damage in rats via the MAPKs/NF-κB and AMPK/Nrf2 signaling pathways. Int J Mol Sci. 2023;24:6900. 37108064 10.3390/ijms24086900 PMC10138912

