# Supplementary material for: Dachaihu decoction alleviates septic liver injury by modulating the intestinal barrier dysfunction and suppressing the NF-κB/NLRP3/Caspase-1 signaling pathway
Source: Chin Med. 2026 Jun 16;21:168. doi: 10.1186/s13020-026-01420-1 (PMC13270745; doi:10.1186/s13020-026-01420-1)
Supplement: Supplementary file 2 — Additional file2 (DOCX 1175 KB) [file 13020_2026_1420_MOESM2_ESM.docx]

Pearson correlation analysis was performed on the test results of DCHD samples, as presented in Figures 1A and 1B. Correlation coefficients greater than 0.9 are generally considered to indicate a strong correlation. The experimental results showed that all inter-sample correlation coefficients exceeded 0.9, demonstrating good experimental repeatability and stable, reliable data.

**
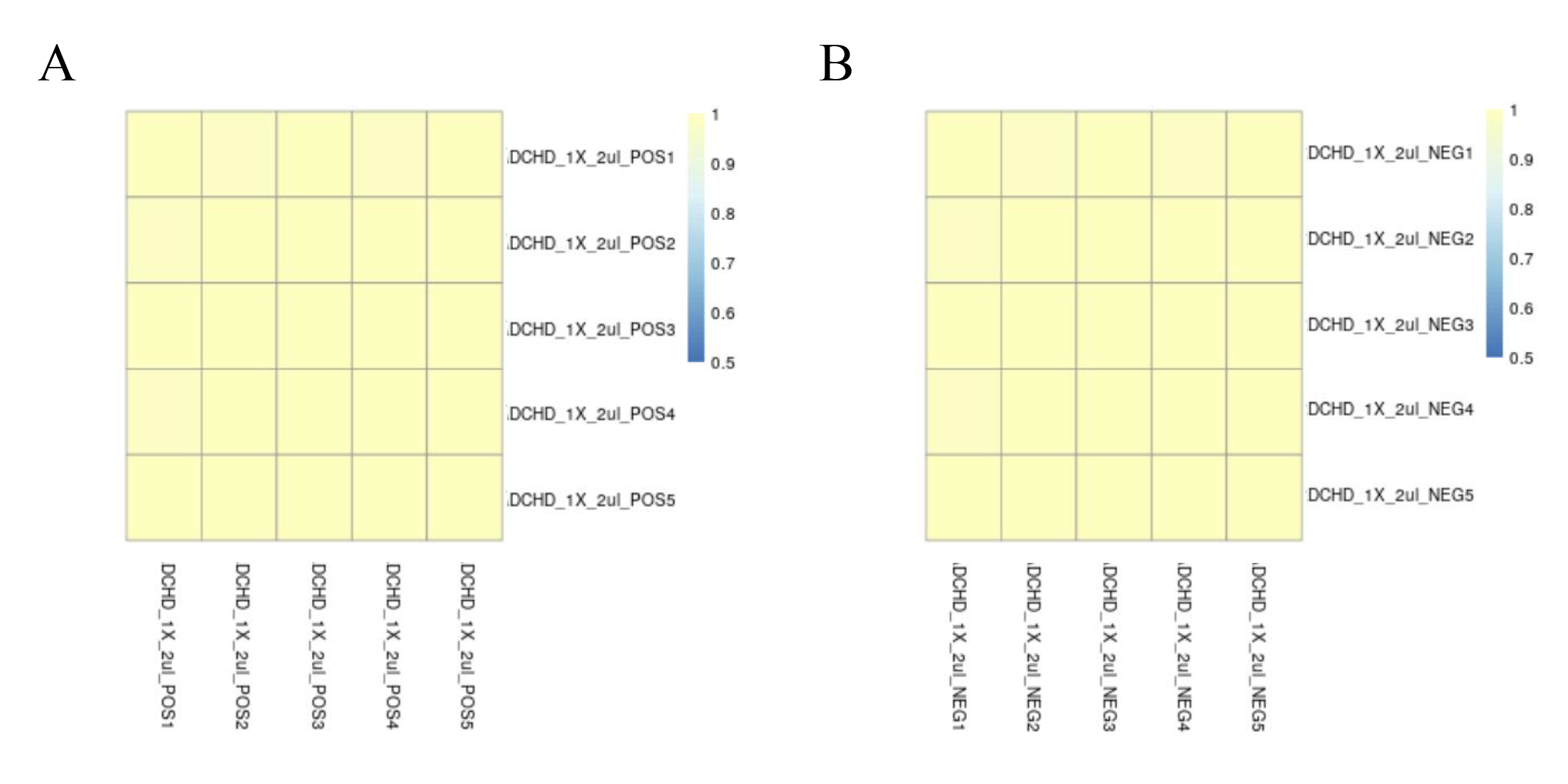
**

Figure 1A. Correlation analysis of traditional Chinese medicine samples in positive ion mode. Figure 1B. Correlation analysis of traditional Chinese medicine samples in negative ion mode. Note: The horizontal and vertical axes represent replicate measurements of the traditional Chinese medicine samples. Each dot in the grid represents an ion peak (compound) extracted from the samples, with the axes corresponding to the logarithmically transformed signal intensities of the ion peaks.

Identification of the chemical constituents of DCHD by HPLC-Q-TOF-MS

| No. | compounds | Molecular formula | m/z | RT [min] | Reference Ion | 2D Structure |
| --- | --- | --- | --- | --- | --- | --- |
| 1 | 2-Hydroxyhippuric acid | C9H9NO4 | 218.04265 | 1.079 | [M+H]+1 | 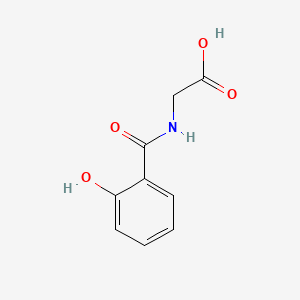 |
| 2 | Trigonelline | C7H7NO2 | 138.05504 | 1.457 | [M+H]+1 | 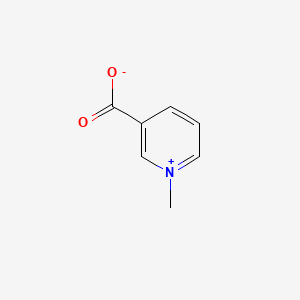 |
| 3 | Theophylline | C7H8N4O2 | 179.05515 | 1.562 | [M-H]-1 | 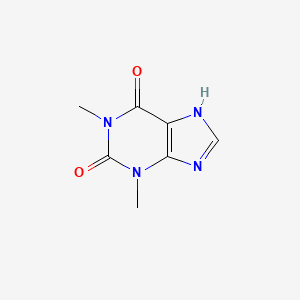 |
| 4 | Citric acid | C6H8O7 | 191.01892 | 2.848 | [M-H+HAc]-1 | 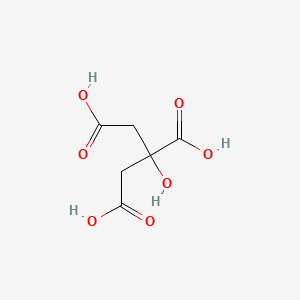 |
| 5 | L-Phenylalanine | C9H11NO2 | 166.0863 | 5.316 | [M+NH4]+1 | 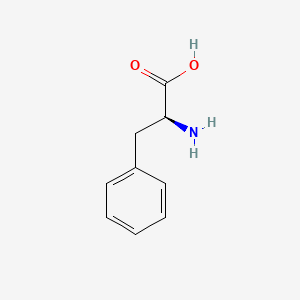 |
| 6 | 3-Ethyl-4-hydroxy-1-phenyl-1,2-dihydroquinolin-2-one | C17H15NO2 | 266.11389 | 7.693 | [M+H]+1 | 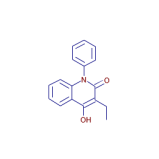 |
| 7 | Coniferin | C16H22O8 | 360.16513 | 9.119 | [M+ACN+H]+1 | 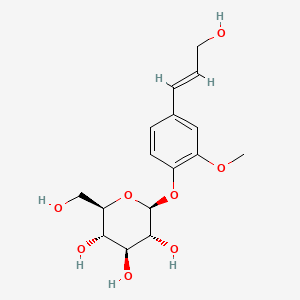 |
| 8 | Salicylic acid | C7H6O3 | 137.02321 | 9.219 | [M-H]-1 | 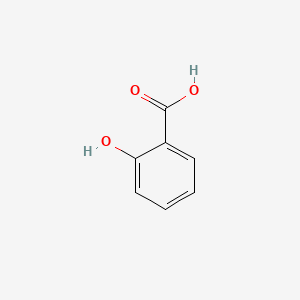 |
| 9 | Albiflorin | C23H28O11 | 525.16119 | 10.126 | [M-H]-1 | 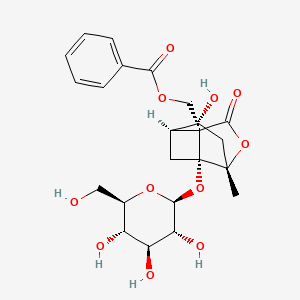 |
| 10 | Quercetin | C15H10O7 | 301.03555 | 11.077 | [M-H]-1 | 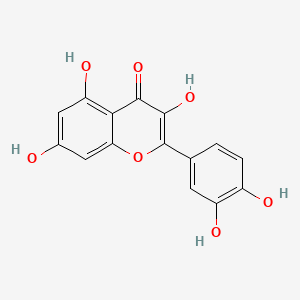 |
| 11 | Esculetin | C9H6O4 | 179.03403 | 11.354 | [M+H]+1 | 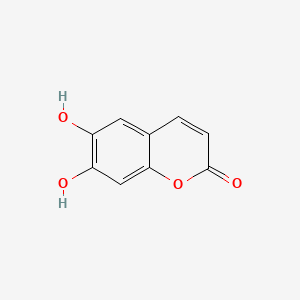 |
| 12 | Hesperetin | C16H14O6 | 303.08615 | 12.001 | [M+H]+1 | 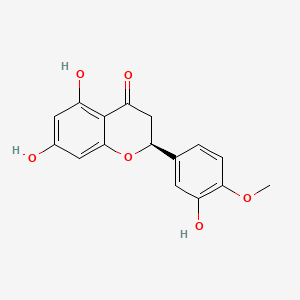 |
| 13 | Genistein | C15H10O5 | 303.08627 | 12.349 | [M+H+MeOH]+1 | 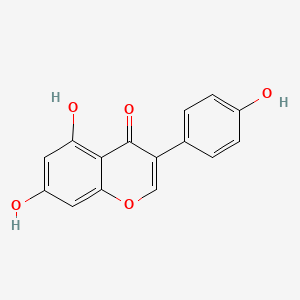 |
| 14 | Naringin | C27H32O14 | 625.17731 | 12.456 | [M+FA-H]-1 | 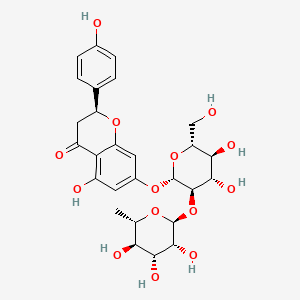 |
| 15 | Kaempferol | C15H10O6 | 287.05469 | 12.609 | [M+H]+1 | 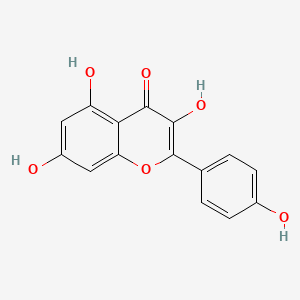 |
| 16 | Nictoflorin | C27H30O15 | 595.16522 | 13.006 | [M+H]+1 | 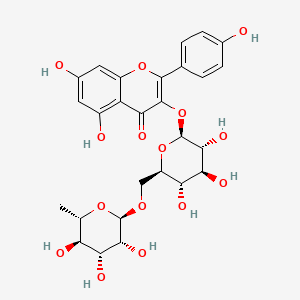 |
| 17 | Neohesperidin | C28H34O15 | 609.18256 | 14.04 | [M-H]-1 | 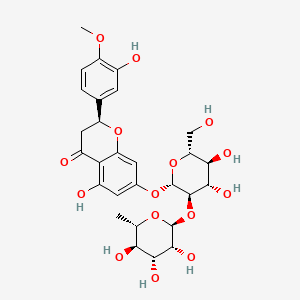 |
| 18 | 6-O-Methylscutellarin | C22H20O12 | 477.10239 | 14.215 | [M+H]+1 | 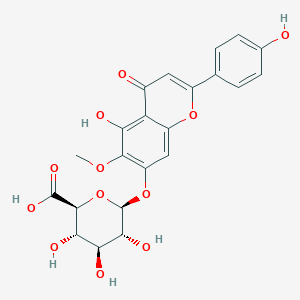 |
| 19 | Baicalin | C21H18O11 | 447.09137 | 14.569 | [M+H]+1 | 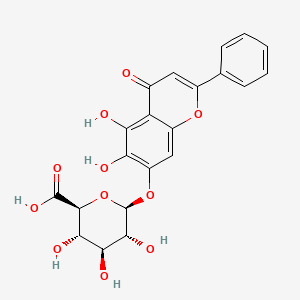 |
| 20 | Apigetrin | C21H20O10 | 433.11316 | 15.162 | [M+H+MeOH]+1 | 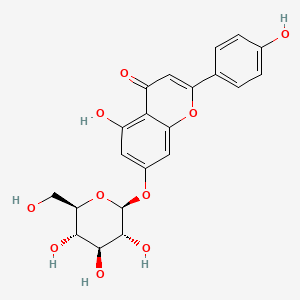 |
| 21 | Wogonin | C16H12O5 | 285.07498 | 15.407 | [M+H]+1 | 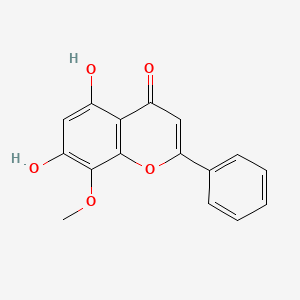 |
| 22 | Obacunone | C26H30O7 | 455.20627 | 16.032 | [M+H]+1 | 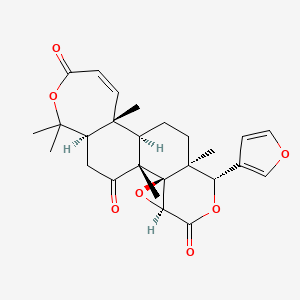 |
| 23 | Tangeritin | C20H20O7 | 373.12778 | 16.312 | [M+H]+1 | 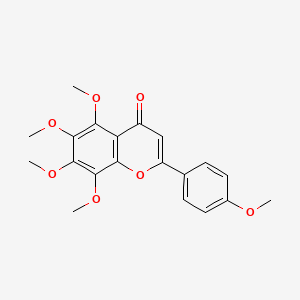 |
| 24 | Rubiadin | C15H10O4 | 253.05045 | 16.535 | [M-H]-1 | 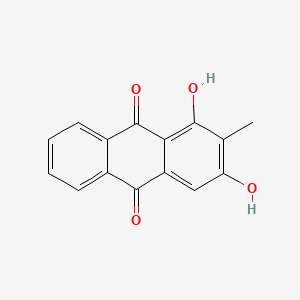 |
| 25 | Shogaol | C17H24O3 | 277.17975 | 17.171 | [M+H]+1 | 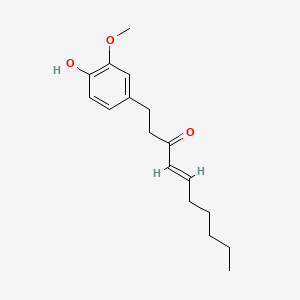 |
| 26 | Nobiletin | C21H22O8 | 403.13867 | 17.77 | [M+H]+1 | 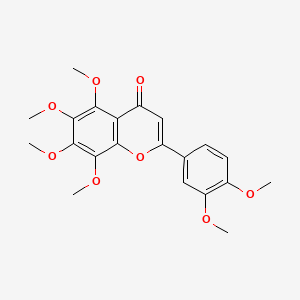 |
| 27 | Saikosaponin A | C42H68O13 | 255.02985 | 17.962 | [M-H]-1 | 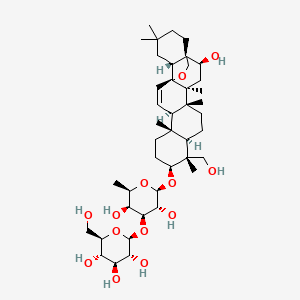 |
| 28 | Genistein | C15H10O5 | 269.04547 | 18.745 | [M-H]-1 | 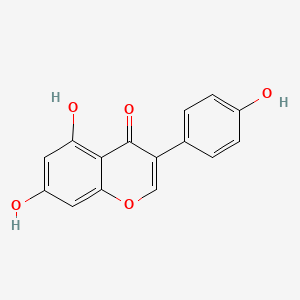 |
| 29 | Daidzein | C15H10O4 | 253.05042 | 19.063 | [M-H]-1 | 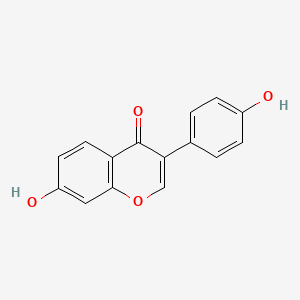 |
| 30 | Muscone | C16 H30O | 239.23677 | 21.924 | [M+H]+1 | 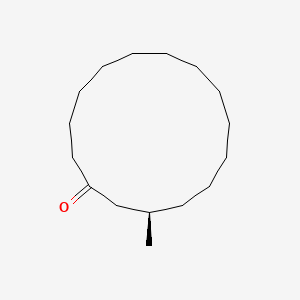 |
| 31 | 1-Stearoylglycerol | C21 H42O4 | 381.29684 | 23.053 | [M+Na]+1 | 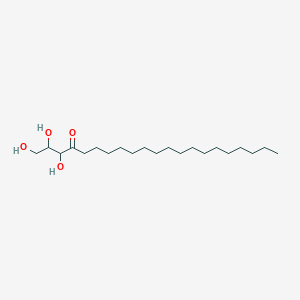 |
| 32 | Emodin | C15H10O5 | 409.32889 | 24.379 | [M+Na]+1 | 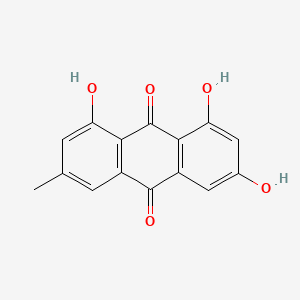 |
