# Supplementary material for: Dachaihu decoction alleviates septic liver injury by modulating the intestinal barrier dysfunction and suppressing the NF-κB/NLRP3/Caspase-1 signaling pathway
Source: Chin Med. 2026 Jun 16;21:168. doi: 10.1186/s13020-026-01420-1 (PMC13270745; doi:10.1186/s13020-026-01420-1)
Supplement: Supplementary file 4 — Additional file4 (DOCX 13 KB) [file 13020_2026_1420_MOESM4_ESM.docx]

**Table S1**

Herb composition in DCHD.

| Chinese name | Latin name | Origin of herb | Medicinal part | Weight (g) | Source |
| --- | --- | --- | --- | --- | --- |
| Chaihu | Bupleuri Radix | *Bupleurum chinense* DC. | Root | 15 | Shanxi, China |
| Huangqin | Scutellariae Radix | Scutellaria baicalensis Georgi | Root | 9 | Shandong, China |
| Dahuang | Rhei Radix et Rhizoma | *Rheum officinale* Baill*.* | Root and rhizome | 6 | Sichuan, China |
| Zhishi | Aurantii Fructus Immaturus | *Citrus aurantium* L. | Fruit | 9 | Hunan, China |
| Banxia | Pinelliae Rhizoma | *Pinellia ternata* Breit. | Tuber | 9 | Gansu, China |
| Baishao | Paeoniae Radix Alba | *Paeonia lactiflora* Pall. | Root | 9 | Anhui, China |
| Shengjiang | Zingiberis Rhizoma Recens | *Zingiber officinale* Roscoe. | Rhizome | 15 | Yunnan, China |
| Dazao | Jujubae Fructus | *Ziziphus jujuba* Mill. | Fruit | 12 | Xinjiang, China |
